# ZnO-Doped gC_3_N_4_ Nanocapsules for Enhancing the Performance of Electroless NiP Coating—Mechanical, Corrosion Protection, and Antibacterial Properties

**DOI:** 10.1021/acsomega.2c07288

**Published:** 2023-06-13

**Authors:** Fatma Nabhan, Eman M. Fayyad, Mostafa H. Sliem, Farah M. Shurrab, Kamel Eid, Gheyath Nasrallah, Aboubakr M. Abdullah

**Affiliations:** † Center for Advanced Materials, Qatar University, Doha, Qatar 2713; ‡ Biomedical Research Center, Qatar University, Doha, Qatar 2713; § Gas Processing Center, Qatar University, Doha, Qatar 2713

## Abstract

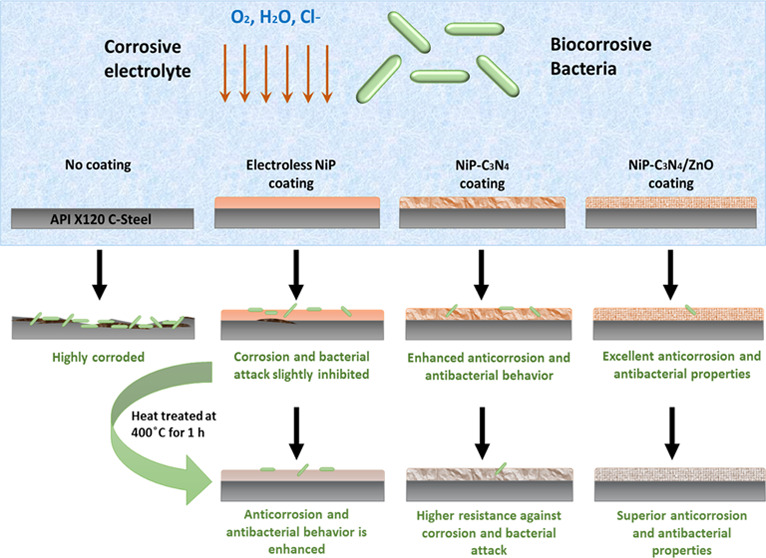

A carbon nitride (C_3_N_4_) nanomaterial has superior mechanical, thermal, and tribological properties, which make them attractive for various applications, including corrosion-resistant coatings. In this research, newly synthesized C_3_N_4_ nanocapsules with different concentrations (0.5, 1.0, and 2.0 wt %) of ZnO as a dopant were incorporated into the NiP coating using an electroless deposition technique. The nanocomposite coatings either ZnO-doped (NiP-C_3_N_4_/ZnO) or undoped (NiP-C_3_N_4_) were heat-treated at 400 °C for 1 h. The as-plated and heat-treated (HT) nanocomposite coatings were characterized by their morphology, phases, roughness, wettability, hardness, corrosion protection, and antibacterial properties. The results indicated that the microhardness of as-plated and heat-treated nanocomposite coatings was significantly improved after the incorporation of 0.5 wt % ZnO-doped C_3_N_4_ nanocapsules. The outcomes of electrochemical studies revealed that the corrosion resistance of the HT coatings is higher than the corresponding as-plated ones. The highest corrosion resistance is achieved on the heat-treated NiP-C_3_N_4_/1.0 wt % ZnO coatings. Although the presence of ZnO in the C_3_N_4_ nanocapsules increased its surface area and porosity, the C_3_N_4_/ZnO nanocapsules prevented localized corrosion by filling the microdefects and pores of the NiP matrix. Furthermore, the colony-counting method used to evaluate the antibacterial behavior of the different coatings demonstrated superior antibacterial properties, namely, after heat treatment. Therefore, the novel perspective C_3_N_4_/ZnO nanocapsules can be utilized as a reinforcement nanomaterial in improving the mechanical and anticorrosion performance of NiP coatings in chloride media, together with providing superior antibacterial properties.

## Introduction

Corrosion is a significant problem that leads to great economic loss and catastrophic failures. The rising demands to enhance corrosion protection methods, namely, coatings, which are commonly used in oil and gas pipelines, automotive equipment, and various engineering applications, have recently encouraged extensive research.^1^ Electroless-deposited nickel phosphorous (NiP) coatings have specifically triggered the researchers’ interest due to their superior hardness, corrosion, and wear resistance.^2−5^ Additionally, electroless NiP coatings will always form uniform deposition on any shape of substrates, even the ones with complicated styles.^6,7^ In an electroless plating technique, NiP is autocatalytically deposited on the substrate using a reducing agent as a source of electrons rather than applying external power.^8^ Despite all of the attractive properties of NiP coatings, researchers are still trying to enhance its overall performance to expand its uses and applications in various aggressive environments. Capacitating the NiP matrix with multiple types of particulates was widely investigated and revealed significant enhancement in the physical, mechanical, corrosion, and wear resistance of the electroless NiP metallic coating.^4^ Many researchers succeeded in improving the properties of electroless NiP coating through the incorporation of, for example, SiC, ZrO_2_, TiO_2_, Al_2_O_3_, ZnO, etc.^9−14^ Fayyad et al. have synthesized a novel NiP-C_3_N_4_ nanocomposite through the codeposition of C_3_N_4_ nanosheets with the NiP matrix, which resulted in a significant enhancement in the microhardness and corrosion resistance properties compared with pure NiP coating.^15^

Carbon nitride (C_3_N_4_) nanomaterials have significant properties, which open the door for many future advancements and applications. It is a very hard material, as hard as diamond, and thermally as well as chemically stable.^16^ Moreover, carbon nitride possesses a novel photocatalytic activity in the visible light region and can be synthesized in different shapes, e.g., nanosheets, nanorods, nanoflowers, nanospheres, etc.^17−21^ Numerous investigations have reported the enhancement of the photocatalytic activity of carbon nitride as the shape of C_3_N_4_ nanoparticles is changed.^22^ Only a few reports have investigated the effect of C_3_N_4_ nanosheets on the physical, mechanical, and corrosion resistance of electroless NiP coatings.^15,23^ Kumar et al. reported improved corrosion resistance performance of pure epoxy (PE) coating using the ZnO–graphitic carbon nitride (ZnO/GCN) nanocomposite as a nanofiller in the PE matrix. The corrosion assessment in their research showed that ZnO/GCN nanosheets have greatly enhanced the surface protective and barrier performance of PE against a corrosive 3.5% NaCl environment.^24^ However, the effect of using different shapes and the nature of the C_3_N_4_ nanomaterial on the corrosion performance of metallic NiP coatings have not been addressed yet. Therefore, in this research, we aim to investigate the incorporation of a new shape of C_3_N_4_ nanomaterials in the electroless NiP coating through the synthesis of C_3_N_4_ nanocapsules without and with different concentrations (0.5, 1.0, and 2.0 wt %) of zinc oxide (ZnO) as a dopant inside the C_3_N_4_ nanocapsules. The influence of the newly fabricated undoped (C_3_N_4_) and doped (C_3_N_4_/ZnO) nanocapsules on the physical, chemical, mechanical, corrosion protection, and antibacterial properties of the NiP-C_3_N_4_ nanocomposite coating was studied. Moreover, this work explored the impact of heat treatment at 400 °C for 1 h on the aforementioned properties of the prepared NiP-C_3_N_4_ nanocomposite coatings.

## Experimental Techniques

### Specimen Pretreatment

API X-120 C-steel was used as a substrate for electroless deposition, as it is a widely used metal in the oil and gas industry. A C-steel bar, with a wt % composition shown in Table 1 below, was cut into 20 × 20 × 10 mm^3^ specimens and pretreated (mechanically and chemically) before electroless deposition.

**Table 1:** Weight % Composition of API X-120 C-Steel

element	C	Mn	V	Si	Cr	Cu	Ni	Mo	Fe
wt %	0.129	0.541	0.025	0.101	0.039	0.015	0.017	0.0013	balance

The pretreatment process included grinding with various emery papers up to 2000 grit and polishing until a mirror-finishing surface was obtained. Other pretreatment steps were also required for the specimens, which included chemical degreasing with acetone for 15 min, followed by alkaline cleaning for 5 min at 80 °C. The alkaline cleaning solution consists of 50 g L^–1^ NaOH, 30 g L^–1^ Na_3_PO_4_, and 30 g L^–1^ Na_2_CO_3_. Subsequently, the specimens were etched in an acidic solution of 15 wt % H_2_SO_4_ for 20 s. After each pretreatment step, the samples were thoroughly washed with ultrapure deionized water to remove contaminants. Finally, the specimens were ready for electroless deposition after the pretreatment steps. Solutions of analytical grade purchased from Sigma-Aldrich (St. Louis, MO) were used for all of the experimental preparations.

### Electroless Deposition

A commercial electroless nickel–phosphorus plating solution (Nichem 3010), purchased from Atotech Deutschland GmbH (Berlin, Germany), was used for the different electroless depositions. A concentration of 0.5 g L^–1^ C_3_N_4_ nanocapsules was kept constant for various prepared electroless NiP-C_3_N_4_ baths. The synthesized C_3_N_4_ nanocapsules, undoped and doped with different concentrations of ZnO, i.e., 0.5, 1.0, and 2.0 wt % ZnO, were separately ultrasonicated for 2 h in the electroless NiP solution before the deposition process. This step was essential to ensure well-dispersed nanomaterial in the bath and avoid agglomeration. The composition and operating conditions of NiP-C_3_N_4_ baths during the electroless deposition process are summarized in Table 2.

**Table 2:** Operational Parameters and Bath Constituents of the Electroless Plating Process

bath constituent		operating parameters	
electroless NiP solution (L)	1	plating temperature (°C)	89 ± 1 °C
amount of g-C_3_N_4_ nanocapsules (g L^–1^)	0.5	stirring (rpm)	300
amount of doped ZnO in C_3_N_4_ (wt %)	0.5, 1.0, and 2.0	plating time (h)	2
		pH	4.5 ± 0.1

After preparing the electroless baths, the pretreated specimens of API X-120 C-steel were instantly immersed in each bath, and the electroless coating process was allowed for 2 h. Finally, the coated specimens were removed from the bath, washed with deionized water, and dried using blowing air.

### Heat Treatment

After the electroless deposition process, samples of the various produced nanocomposite coatings were annealed at 400 °C for 1 h using a vacuum tube furnace from MTI (California). This step was essential to investigate the different properties of the prepared nanocomposite coatings after heat treatment.

### Characterization

The crystal structure, surface morphology, elemental composition, roughness, hydrophobicity, mechanical, anticorrosive, and antibacterial properties of the different prepared coatings were investigated before and after heat treatment using various characterization techniques.

#### Structural Analysis X-ray Diffractometer (XRD)

The effectiveness of the undoped C_3_N_4_ nanocapsules, in addition to changing ZnO dopant concentrations in the C_3_N_4_ nanocapsules on the crystal structure and phases of the electroless NiP and NiP-C_3_N_4_ nanocomposite coatings, respectively, was analyzed using an X-ray diffractometer (XRD, Miniflex2 Desktop, Cu K, Rigaku, Tokyo, Japan).

#### Surface Morphology and Compositional Analysis

Scanning electron microscopy (SEM, Nova NanoSEM 450, Thermo Fisher Scientific, Eindhoven, Netherlands) was used to assess the surface morphology and coating thickness of each sample either as-plated or heat-treated. Energy-dispersive X-ray spectroscopy (EDX, Bruker detector 127 eV, Bruker, Leiderdorp, Netherlands) was utilized to achieve the elemental analysis of the different prepared coatings. To get an image, the microscope was operated at 200 kV.

#### Surface Roughness and Water Contact Angle (WCA)

Atomic force microscopy (AFM) test for the synthesized coatings before and after heat treatment was performed by operating an MFP3D Asylum research atomic force microscope (Asylum Research, Santa Barbara, CA), which was equipped with a silicon probe. The roughness experiments were conducted under a spring constant of 2 Nm^–1^ and a resonant frequency of 70 kHz, and the device was run under ambient conditions using the tapping mode in air.

The water contact angle (WAC) measurements for the as-plated and heat-treated specimens were performed to identify the level of hydrophobicity and hydrophilicity of prepared coatings. This technique was achieved utilizing a contact angle device (DataPhysics OCA35, DataPhysics Instruments GmbH, Filderstadt, Germany). The used probing liquid was deionized water (4 μL), and for achieving precise measurements, the contact angle was measured 5 times, where the average value was reported.

#### Mechanical Analysis

The microhardness of the various coatings (before and after heat treatment) was measured using a Vickers microhardness tester (FM-ARS9000, Future- Tech Corp., Tokyo, Japan). An average value of five microhardness measurements was calculated for each sample at 200 g of load for 10 s. Additionally, the mechanical characterization of the different coatings was further emphasized using the nanoindentation test. The measurements were obtained using a nanoindenter head connected to an AFM device at 1 mN maximum indentation force, 200 μN/s loading and unloading rates, and 5 s dwell time.

#### Corrosion Study

To study the corrosion resistance of the prepared NiP and NiP-C_3_N_4_ nanocomposite coatings and to investigate the effect of ZnO concentrations (as a dopant) on the corrosion protection properties, electrochemical impedance spectroscopy (EIS) and potentiodynamic polarization (PP) (Tafel test) were carried out in a solution of 3.5 wt % sodium chloride (NaCl) at room temperature. The corrosion resistance measurements were done for all coated samples before and after heat treatment. All corrosion measurements were carried out by utilizing a GAMRY 3000 potentiostat/galvanostat/ZRA device (Warminster, PA) that was connected to a three-electrode cell, in which the coated substrate was the working electrode, and the reference and counter electrodes were selected to be Ag/AgCl and graphite rod, respectively. The EIS test was run at an excitation of 10 mV AC, a frequency range between 1 × 10^–2^ and 1 × 10^5^ Hz, and the open-circuit potential was always allowed to stabilize before the recording of EIS data started. On the other hand, the Tafel tests were performed to obtain anodic and cathodic polarization curves by applying a scan rate of 0.167 mV s^–1^ within the initial and final potential of −250 and 250 mV, respectively. The measurements were repeated three times to ensure reproducibility.

#### Antibacterial Study

The antibacterial test was used to investigate the antibacterial properties of the prepared nanocomposite coatings before and after heat treatment, which could be achieved using the so-called colony-counting method. The prepared specimens of C-steel coated with NiP-C_3_N_4_ nanocomposite coatings using undoped and doped C_3_N_4_ nanocapsules with different concentrations of ZnO (i.e., 0.5, 1.0, and 2.0 wt %, respectively) were sterilized using 70% ethanol. Using glue and DPX mountant, specimens were glued into six-well plates from the lower side where it was not coated. Then, the plate with the specimen was sterilized under UV light for 30 min. A bacterial culture of *Staphylococcus aureus* (*S. aureus*) was allowed to grow in LB broth until the OD at 600 nm reached the late log phase (1.2 OD). Then, 5 mL of bacterial broth was added to each well having the coated specimen. The plate was incubated in a shaker at 37 °C at 50 RPM for 3 h. After 3 h, the LB was removed, and all specimens were washed with autoclaved distilled water 2 times. After washing, the coated specimens were scraped using a loop and 2 mL of autoclaved distilled water to remove any bacterial growth if found. Then, the 2 mL scrapped bacteria with DW was transferred to an Eppendorf tube. A 1:10 serial dilution was performed, then 100 μL from the 1:1000 dilution was cultured in an agar plate and incubated at 37 °C overnight. The next day, bacterial colonies were counted on each plate. Then, the average values for the obtained number of colonies for all assays were used to calculate the antibacterial performance (*R*) of the prepared samples, using the following equation
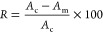
where *A*_c_ and *A*_m_ are the number of colonies obtained by the detached bacterial suspension obtained by the control C-steel and the coated samples, respectively.

## Results and Discussion

### Structural Analysis (XRD)

**Figure 1:**
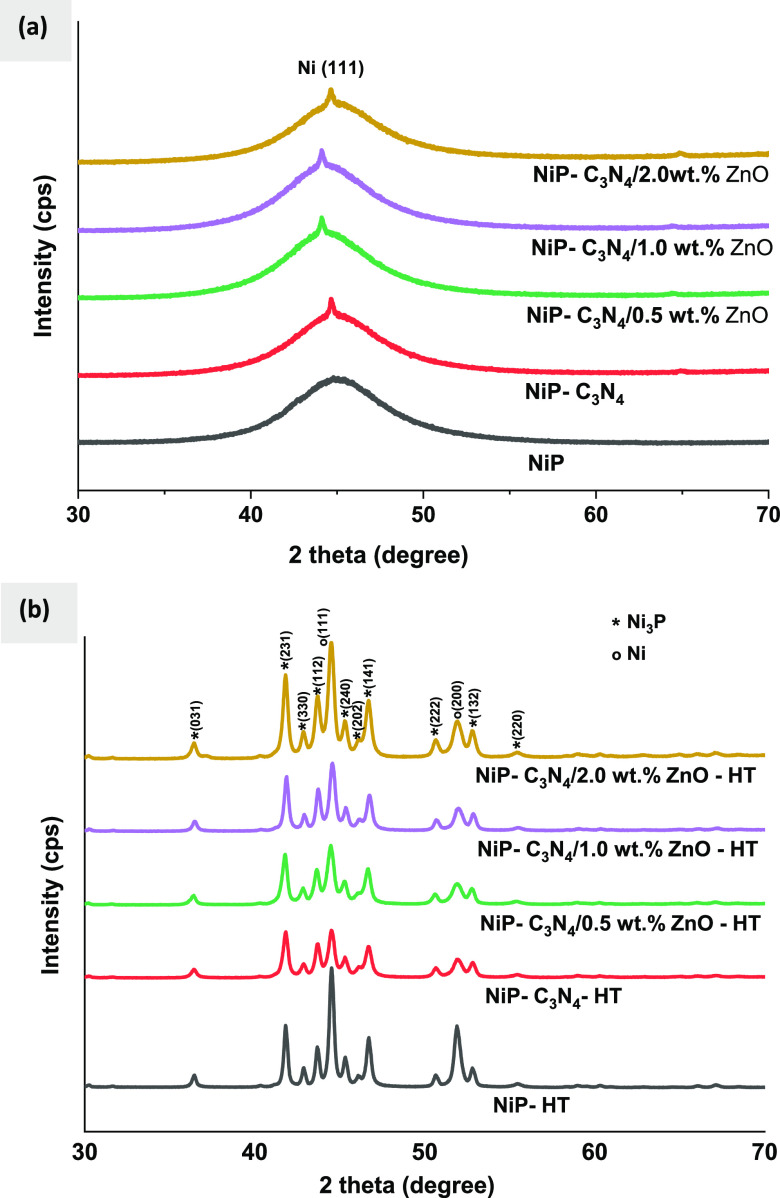
XRD pattern of NiP, NiP-C_3_N_4_, and NiP-C_3_N_4_/ZnO with different concentrations of ZnO (0.5, 1.0, and 2.0 wt %) (a) before and (b) after heat treatment at 400 °C for 1 h.

Figure 1a,b represents the XRD patterns obtained for electroless NiP, NiP-C_3_N_4_, and NiP-C_3_N_4_/ZnO nanocomposite coatings with different concentrations (0.5, 1.0, and 2.0 wt %) of doped ZnO in C_3_N_4_ nanocapsules before and after heat treatment, respectively. It can be seen in Figure 1a that the as-plated NiP coating has a broad peak extended at the 2θ position = 45°. This single broad peak is associated with the (111) plane of the face-centered cubic (FCC) Ni. Moreover, all of the other nanocomposite coatings show the same single broad peak at the same position, i.e., 2θ = 45°, and no other peaks that identify C_3_N_4_ appear. This might be due to the acceptable size and small amounts of the incorporated C_3_N_4_ nanocapsules compared to the high density of the Ni diffraction peaks of the NiP coating. Several researchers observed a similar result when a small amount of nanomaterial was incorporated into the NiP matrix.^25,26^ Additionally, it can be noticed that after the incorporation of undoped or ZnO-doped C_3_N_4_ nanocapsules in the NiP coating, the structure changes from an amorphous to a crystalline-amorphous or semiamorphous state. According to the literature,^27^ the *P* content determines the crystal structure of the NiP coating, i.e., high *P* content (≥10 wt %) gives amorphous structure, whereas medium (5–10 wt %) or low (1–5 wt %) *P* content leads to a semicrystalline or crystalline structure, respectively. Since the XRD patterns show amorphous and semicrystalline structures for C_3_N_4_-free and undoped/doped C_3_N_4_ coatings, respectively, the high and medium *P* contents in these coatings are verified, which is in line with the EDX results. Furthermore, the XRD patterns reveal full width at half-maximum (FWHM) values of 0.6140, 0.5178, 0.5140, 0.5145, and 0.5130 for NiP, NiP-C_3_N_4_, and NiP-C_3_N_4_ that are doped 0.5, 1.0, and 2.0 wt % with ZnO nanocomposite coatings, respectively. As a result, the refinement of the NiP nodules and the boosting of the crystalline phase formation are achieved in the occurrence of undoped and doped C_3_N_4_ in the NiP matrix. Several studies confirmed that the structure of electroless NiP coating changes from an amorphous state to a crystalline form by heat treatment,^28^ which is compatible with the XRD patterns, as shown in Figure 1b, obtained for NiP, NiP-C_3_N_4_, and NiP-C_3_N_4_/ZnO with different ZnO concentrations of nanocomposite coatings after heat treatment. It can be seen that after heat treatment (HT), NiP crystallizes, forming Ni_3_P particles and crystalline Ni phases on the surface of the NiP coating. Additionally, it can be observed that the diffraction peaks of heat-treated undoped and doped NiP-C_3_N_4_ nanocomposite coatings match those obtained for the HT-NiP coating with a minor change in the intensity of the resulted peaks, as it slightly decreases for all HT nanocomposite coatings, except for the NiP-C_3_N_4_/2.0 wt % ZnO coating. The reduced peaks’ intensity in most of the nanocomposite coatings could have happened due to the decrease in deposited Ni and P that occurs upon the incorporation of undoped or doped C_3_N_4_ nanocapsules, which are well dispersed in the NiP matrix, as shown in the SEM results. On the other hand, the agglomeration of the doped C_3_N_4_ with 2.0 wt % ZnO nanocapsules in the NiP matrix, as shown in SEM, results in having less or almost no effect on the amount of deposited Ni and P over a wide area of the substrate as a result of the measurements possibly being taken from such sites.

### Morphology and Compositional Analysis

#### SEM Analysis

**Figure 2:**
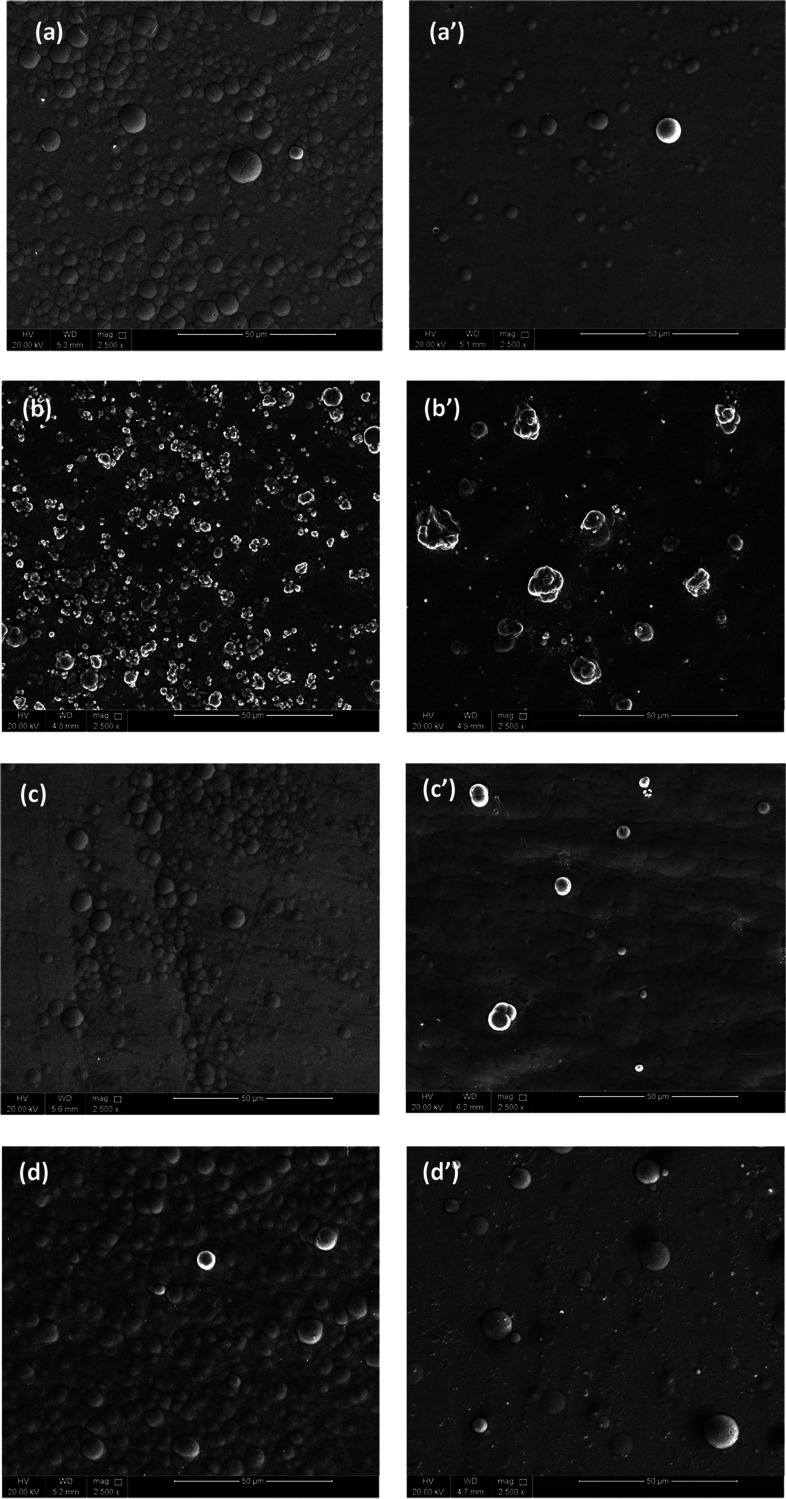

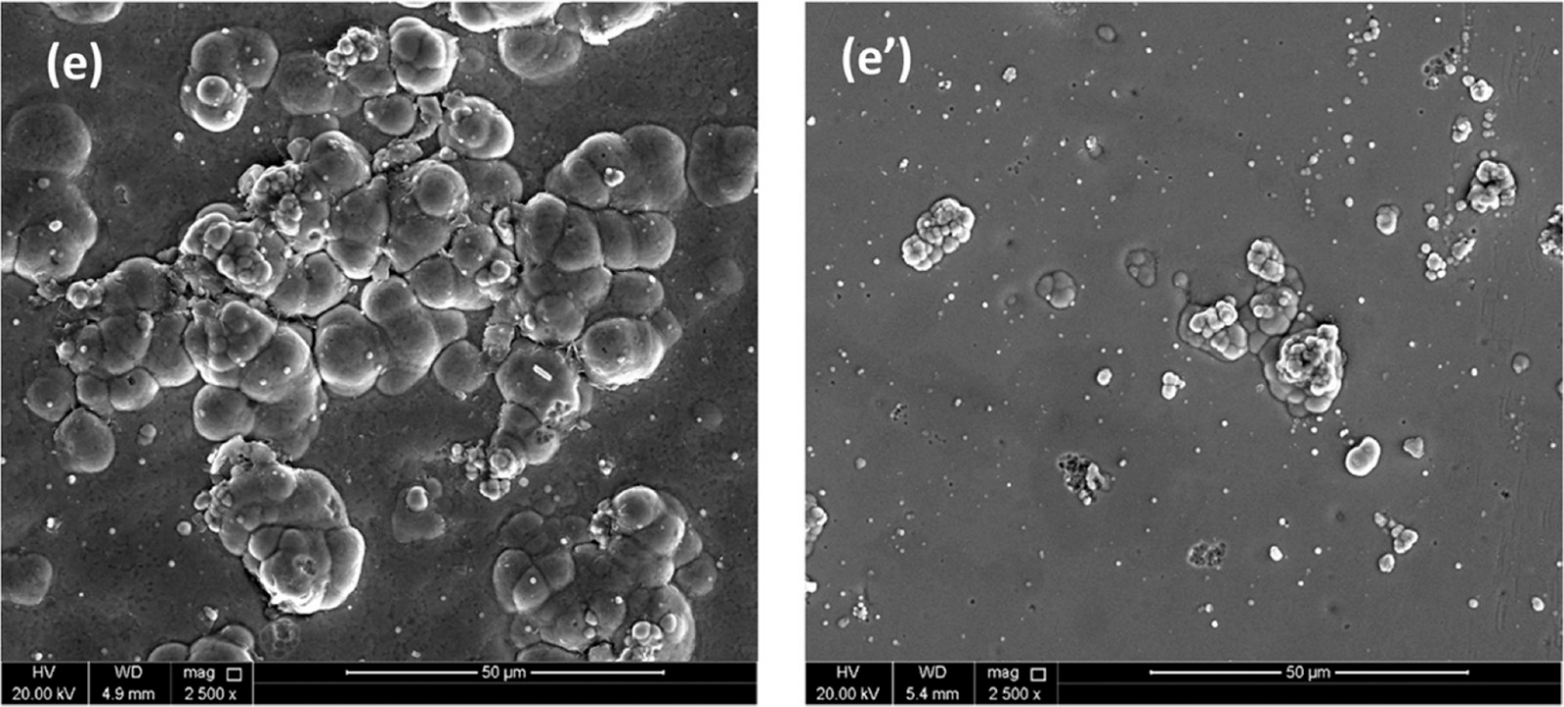
SEM images of (a, a’) NiP, (b, b’) NiP-C_3_N_4_, (c, c’) NiP-C_3_N_4_/0.5 wt % ZnO, (d, d’) NiP-C_3_N_4_/1.0 wt % ZnO, and (e, e’) NiP-C_3_N_4_/2.0 wt % ZnO before and after heat treatment at 400 °C for 1 h.

The SEM images shown in Figure 2a–e and a’–e’ represent the surface morphology of as-plated and heat-treated NiP, NiP-C_3_N_4_, and NiP-C_3_N_4_/ZnO coatings doped with different concentrations of ZnO, i.e., 0.5, 1.0, and 2.0 wt %, respectively. The SEM image of NiP coating shown in Figure 2a indicates a structure like a cauliflower that is characterized by a spherical nodular feature, which is similar to the morphology of electroless NiP reported in several works.^29,30^ Incorporating C_3_N_4_ nanocapsules into the NiP matrix does not change the cauliflower shape of the NiP coating; however, it has a considerable effect on the nodules’ size and arrangement to some extent. As demonstrated in Figure 2b, which corresponds to the as-plated NiP-C_3_N_4_ coating, the surface becomes less smooth, and the size of the nodules significantly decreases while their numbers increase. The morphology change confirms that the C_3_N_4_ nanocapsules are effectively incorporated into the matrix. Various reports in the literature^2,15,25^ indicated a significant morphology change after the incorporation of other types of nanomaterials in the NiP matrix. Doping the C_3_N_4_ nanocapsules with ZnO at different concentrations remarkably affects the surface morphology of the nanocomposite coatings, as illustrated in Figure 2c–e. It is noticeable that the concentration of doped ZnO is increased up to 1.0 wt %, the spherical nodules become more homogeneous and compact, which seems outstanding in NiP-C_3_N_4_/1.0 wt % ZnO. However, the doped ZnO concentration in the C_3_N_4_ nanocapsules is further increased to 2.0 wt %, and some agglomeration of nanocapsules emerges on the surface, which significantly affects the uniformity of the coating. Therefore, its homogeneity and compactness are decreased considerably compared to those of 0.5 and 1.0 wt % ZnO-doped NiP-C_3_N_4_/ZnO nanocomposite coatings. Furthermore, some pores and voids appear on the surface of the NiP-C_3_N_4_/2.0 wt % ZnO composite coating, which extremely affects the corrosion resistance properties of the coating, as will be discussed later. The morphological change of NiP-C_3_N_4_/ZnO nanocomposite coatings is induced by modifying the nanocapsules’ features as the doped ZnO concentration increases. As proved in various studies,^31^ increasing the concentration of doped ZnO in the C_3_N_4_ nanomaterial significantly increases its surface area and porosity. Moreover, other studies^32,33^ revealed distinctive morphological changes in C_3_N_4_ nanomaterials upon using different concentrations of doped ZnO.

After heat treatment, the number of nodules significantly decreases, and the globular morphology in all of the coatings relatively diminishes compared to the corresponding as-plated coatings. The morphology change of the coatings after heat treatment is mainly related to the formation of new phases, as illustrated in XRD analysis. Accordingly, the typical nodular structure of the electroless NiP deposits gets extremely fine as the formed intermetallic Ni_3_P particles are characterized by the crystalline structure. Generally, this occurs due to the change of grain size upon the occurrence of the intermetallics showing different morphologies.^34^ It is also noticeable that the size of the nodules generally decreases, and their numbers increase in an ordered manner, which in turn fosters the smoothness of the surface. Generally, the surface homogeneity and compactness are enhanced after heat treatment for all of the coatings, namely, for HT-NiP-C_3_N_4_/1.0 wt % ZnO. However, at a high concentration of doped ZnO, i.e., 2.0 wt %, the coating surface appears rough, bumpy, and agglomerated compared to those containing 0.5 and 1.0 wt %. This might be attributed to the high porosity of the nanocapsules induced by the high concentration of doped ZnO, as noticed from Brunauer–Emmett–Teller (BET) results shown in Figure S1, which reduces the overall compactness of the coating with the newly formed phases after heat treatment.

#### EDX Analysis

Table 3 summarizes the data obtained from the EDX spectra for the different as-plated and heat-treated coatings. EDX spectra signal the presence of C_3_N_4_ nanocapsules in the NiP coating, as the Ni, P, C, and N peaks appeared, confirming their successful incorporation in the coating. The N peak represents the C_3_N_4_ nanocapsules. In addition, it is observed that the percentage of the P and Ni contents in the NiP-C_3_N_4_ nanocomposite coating is decreased. This can be attributed to adding the codeposited C_3_N_4_ nanocapsules in the coatings. Moreover, the EDX results of the as-plated coatings show that the phosphorus (P) content is significantly decreased by about 50% after incorporating C_3_N_4_ nanocapsules, i.e., from 18.90 to 9.63 wt % P in as-plated NiP and NiP-C_3_N_4_, respectively. This confirms that the structure of as-plated coatings changes from amorphous to semicrystalline, which is consistent with the XRD outcomes. On the other hand, traces of zinc element (Zn), which is related to the doped ZnO in the nanocapsules, appear only in the EDX spectra of NiP-C_3_N_4_/ZnO at high concentrations of ZnO, i.e., 1.0 and 2.0 wt %. This is expected as the ZnO nanoparticles are embedded and reacted within the carbon nitride nanostructures, which hardly can be detected by EDX analysis of the coatings, namely, at low concentrations of ZnO. Since the concentration of either undoped or doped C_3_N_4_ nanocapsules added to the electroless bath is fixed (0.5 g/L), it is depicted that increasing the concentration of doped ZnO in the different nanocomposite coatings has a slight effect on the overall compositions of C, N, and P content. The slight changes are mainly related to the variations in the handling process during preparation. However, it is illustrated that the percentages of O in the composite coatings increased, which may be attributed to the increase of the surface area and the porosity of the C_3_N_4_ nanocapsules, reflecting on the percentage change of the other elements like Ni. After heat treatment, the P content slightly decreases in all of the EDX spectra of the HT coatings, which is mainly attributed to the formation of new phases, such as Ni_3_P, as well as the improved crystallinity of the prepared coatings, which confirms the XRD results.

**Table 3:** EDX Analysis for the Elemental Composition of the Different As-Plated and Heat-Treated Coatings

		before HT		after HT	
coating name	element	norm. C (wt %)	atom. C (atom %)	norm. C (wt %)	atom. C (atom %)
NiP	Ni	81.10	69.36	86.50	77.17
	P	18.90	30.64	13.50	22.83
NiP-C_3_N_4_	Ni	65.47	51.21	66.19	48.74
	P	09.93	16.90	06.11	11.95
	C	15.54	17.19	17.70	24.01
	N	08.70	14.70	10.00	15.30
NiP-C_3_N_4_ (0.5 wt % ZnO)	Ni	60.03	36.05	44.21	35.68
	P	09.23	15.60	06.50	11.03
	C	13.32	19.74	25.47	26.62
	N	09.00	15.48	14.62	16.73
	Zn	00.00	00.00	00.00	00.00
	O	8.42	13.13	09.18	11.94
NiP-C_3_N_4_ (1.0 wt % ZnO)	Ni	56.04	36.77	45.19	26.77
	P	9.59	14.76	06.11	09.89
	C	15.17	16.96	26.72	30.32
	N	9.61	18.86	14.60	22.00
	Zn	00.29	00.30	0.12	00.29
	O	09.30	12.35	08.07	10.73
NiP-C_3_N_4_ (2.0 wt % ZnO)	Ni	51.22	35.27	44.86	31.60
	P	09.11	13.04	05.79	07.93
	C	17.51	21.00	25.67	33.32
	N	9.710	13.03	13.77	15.65
	Zn	0.330	00.38	00.22	00.35
	O	12.12	17.28	09.69	11.15

#### Cross-sectional Analysis

**Figure 3:**
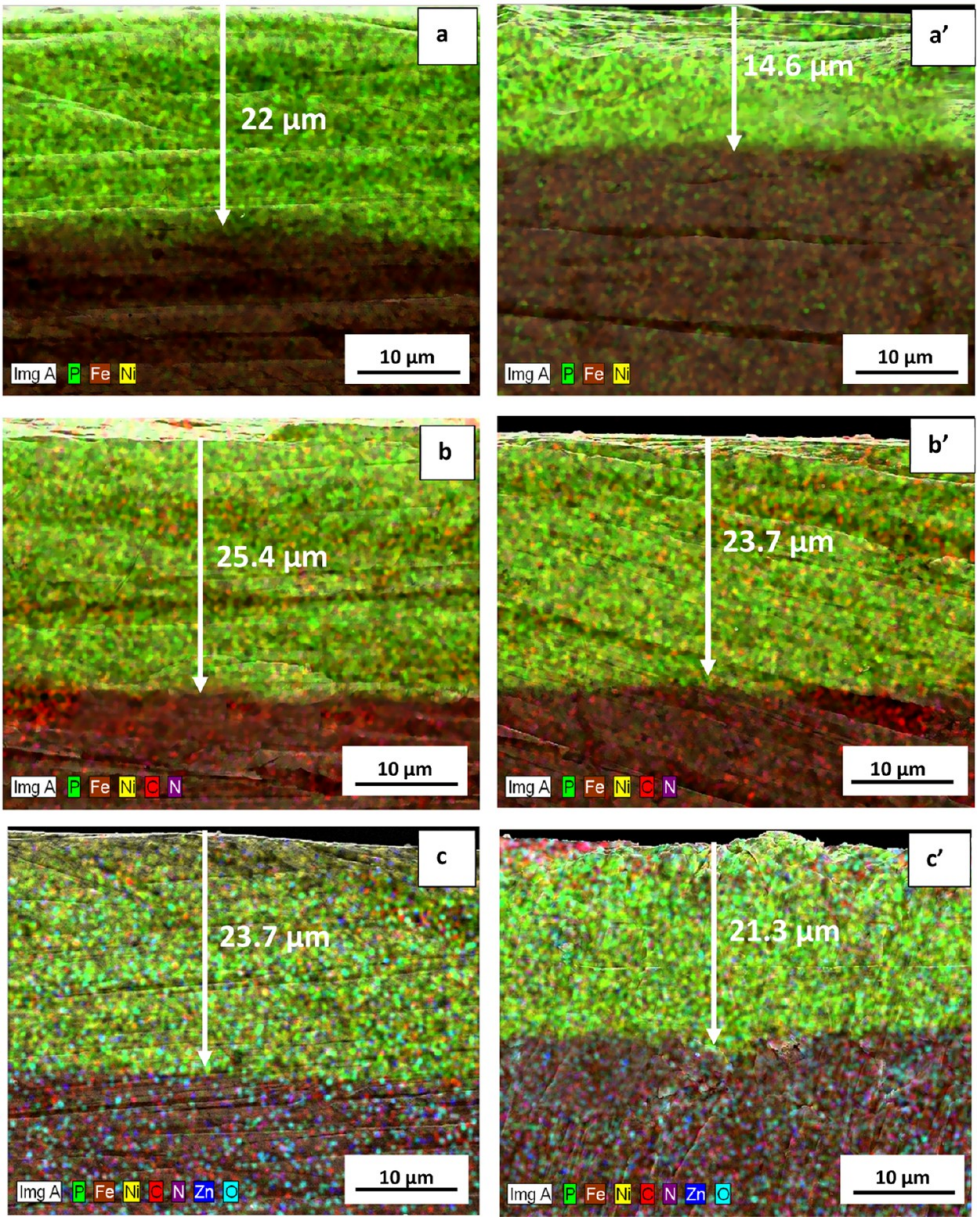
Cross-sectional SEM/EDX image mapping of (a, a’) NiP, (b, b’) NiP-C_3_N_4_, and (c, c’) NiP-C_3_N_4_/1.0 wt % ZnO coatings before and after heat treatment, respectively.

The cross-sectional morphology and EDX mapping of the constituent elements for the as-plated and heat-treated NiP, NiP-C_3_N_4_, and NiP-C_3_N_4_/1.0 wt % ZnO (as a representative for other NiP-C_3_N_4_/ZnO nanocomposite coatings) are shown in Figure 3(a–c) and (a’–c’), respectively. The cross-sectional micrographs show that all coatings are uniform and have no cracks or defects at the interface between the substrate and the coating, elucidating good coating adhesion.^35^ In general, coating adhesion is attained mainly from the uniform distribution of nanoparticles by filling the coating porosities with g-C_3_N_4_ nanoparticles.^36^ Notably, the thickness of the NiP coating (22 μm) is smaller than those of NiP-C_3_N_4_ and NiP-C_3_N_4_/1.0 wt % ZnO coatings, with thicknesses of 25.4 and 23.7 μm, respectively. Accordingly, the increased thickness indicates the induced effect upon incorporating C_3_N_4_ nanocapsules in the NiP matrix. Previous studies^25^ reported that introducing some nanomaterials in the NiP matrix act as a catalytic surface leading to the acceleration of deposition rate. Hence, the coating thickness is increased. Moreover, doping the C_3_N_4_ nanocapsules with ZnO causes a slight decrease in the thickness of nanocomposite coatings, which is indicative of the improved homogeneity of the coating’s morphology. Additionally, the SEM/EDX mapping images of the cross section for all coatings clearly show a uniform distribution and homogeneity of all of the constituent elements, as shown in Figure 3, and confirm that carbon nitride nanocapsules are well dispersed within the thickness of the nanocomposite coatings. After heat treatment, the thickness of the NiP coating considerably decreases from 22 μm to 14.6 μm, as illustrated in Figure 3, which is mainly attributed to the formation of new phases and modified crystallographic structure. As previously demonstrated in the XRD analysis section, after heat treatment, the structure of NiP coating changes from amorphous to semicrystalline, which has a more ordered and uniform structure that is consistent with the EDX results and confirms them. On the other hand, only a slight decrease is indicated in the thickness of the nanocomposite coatings, i.e., NiP-C_3_N_4_ and NiP-C_3_N_4_/1.0 wt % ZnO after heat treatment, which is mainly reduced by around 2 μm. This indicates the incredible effect of the C_3_N_4_ nanocapsules (doped and undoped with ZnO) on the enhancement of thicknesses of the coatings.

#### Transmission Electron Microscopy (TEM) Analysis

**Figure 4:**
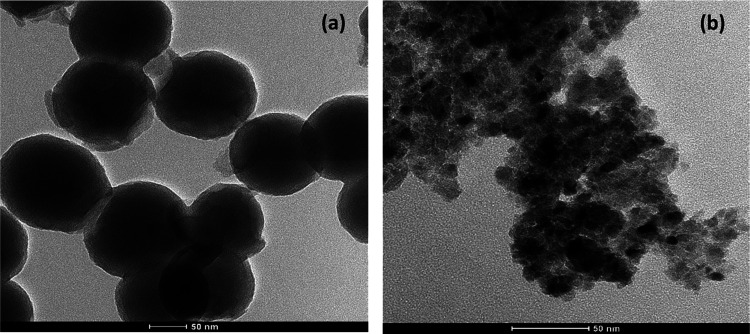
TEM images of (a) carbon nitride (C_3_N_4_) nanocapsules and (b) electroless coating of NiP-C_3_N_4_.

Figure 4a,b demonstrates the TEM images of carbon nitride (C_3_N_4_) nanocapsules and the NiP-C_3_N_4_ nanocomposite coating. It can be seen in Figure 4a the successful preparation of the capsule-shaped carbon nitride nanomaterial. All ZnO-doped and undoped carbon nitride nanocapsules show a similar shape and morphology, except for the 2.0 wt % ZnO-doped-C_3_N_4_ nanocapsules reveal a distorted oval-like morphology due to the increased ZnO concentration. The TEM images of all prepared ZnO-doped and undoped C_3_N_4_ nanocapsules are provided in Figure S2. On the other hand, Figure 4b confirms the excellent distribution of undoped C_3_N_4_ nanocapsules in the NiP matrix. Doping the nanocapsules with 0.5 and 1.0 wt % ZnO does not affect the homogeneous distribution of the nanocapsules in the NiP matrix. However, at a high concentration of doped ZnO, i.e., 2.0 wt %, the modified shape of the carbon nitride nanocapsules leads to its agglomeration in the NiP matrix.

### Surface Roughness (AFM) and Water Contact Angle Measurements (WCA)

#### AFM Analysis

**Figure 5:**
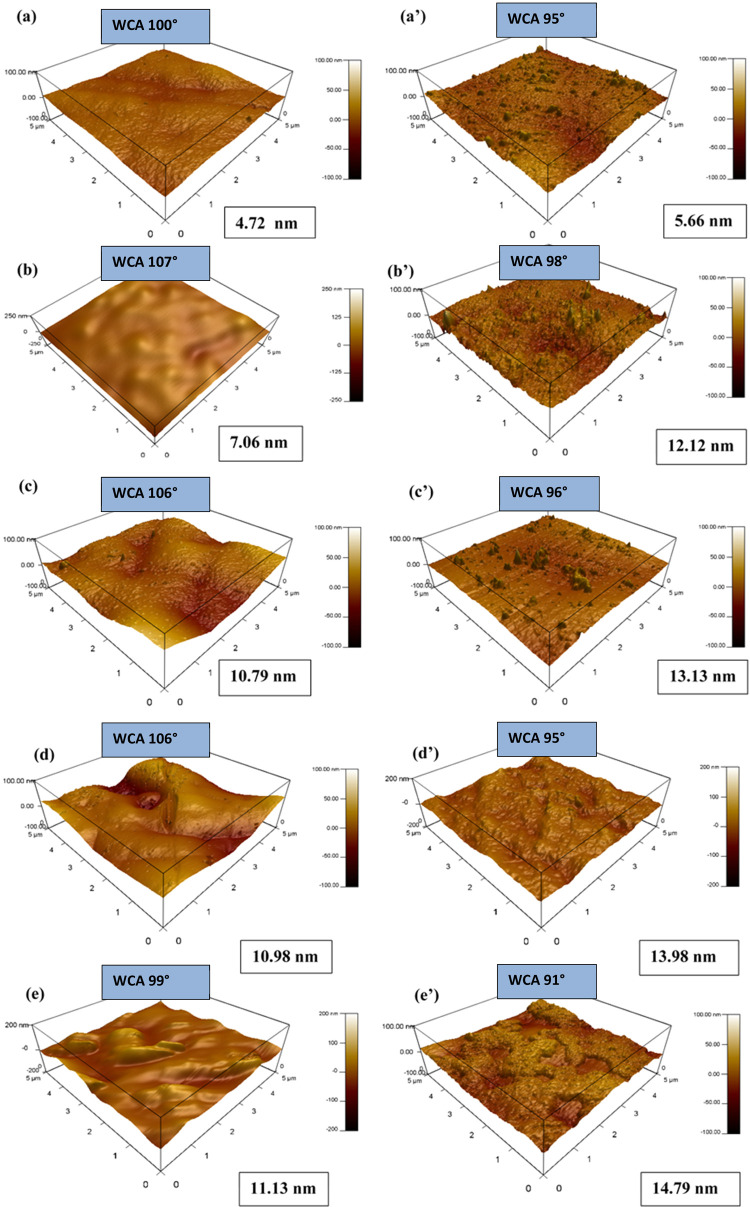
AFM images of (a, a’) NiP, (b, b’) NiP-C_3_N_4_, (c, c’) NiP-C_3_N_4_/0.5 wt % ZnO, (d, d’) NiP-C_3_N_4_/1.0 wt % ZnO, and (e, e’) NiP-C_3_N_4_/2.0 wt % ZnO coatings before and after heat treatment at 400 °C for 1 h, respectively.

Figure 5 shows three-dimensional (3D) AFM images with a measured surface roughness for the different as-plated and heat-treated coatings obtained by the atomic force microscopy (AFM) technique. It is noticed that the surface roughness of as-plated NiP-C_3_N_4_ is higher than that of the C_3_N_4_-free coating by around 3 nm, which reveals that the incorporation of C_3_N_4_ nanocapsules in the NiP matrix leads to an increase in the surface roughness of the plain coating. Moreover, as shown in Figure 5c,e, it is depicted that the surface roughness of the doped C_3_N_4_ nanocomposite coatings slightly increases compared to that of the undoped one. In addition, increasing the concentration of ZnO in the C_3_N_4_ nanocapsules from 0.5 to 1.0 wt % shows a negligible effect on the surface roughness, whereas at a high concentration of doped ZnO, i.e., 2.0 wt %, the surface roughness of the coating increases. The slight increase in the surface roughness corresponds to NiP-C_3_N_4_/2.0 wt % ZnO can be attributed to the distorted morphology and agglomeration of the C_3_N_4_ nanocapsules upon increasing the concentration of doped ZnO. This result is consistent with the TEM and SEM outcomes. Furthermore, it is noticeable that the NiP coating shows only a slight increase in surface roughness (less than 1 nm) after heat treatment. In contrast, it remarkably increases for all of the nanocomposite coatings. This indicates the pronounced effect of the incorporated, doped, and undoped C_3_N_4_ nanocapsules on the roughness properties of the coatings, even after heat treatment. The surface roughness results are, to some extent, contrary to the XRD and SEM results, which prove the more compact and ordered morphology of the nanocomposite coatings after heat treatment. However, it can be said that the increased roughness after heat treatment has a negligible effect on the overall properties of the coatings.

#### WCA Measurements

The WCA measurements determine the degree of hydrophobicity or hydrophilicity of coatings, i.e., hydrophilic surfaces exhibit a small water contact angle (WCA < 90°). In contrast, a large contact angle (WCA > 90°) indicates a hydrophobic surface.^37^ As mentioned in Figure 5, the contact angle of NiP is 100°, which reveals hydrophobic behavior. Similarly, Karthikeyan et al.^38^ reported the hydrophobic behavior of electroless NiP coating. However, the contact angle value increases to 107° for NiP-C_3_N_4_, indicating that the coating surface’s hydrophobic behavior increases after incorporating C_3_N_4_ nanocapsules. On the other hand, doping the C_3_N_4_ nanocapsules with ZnO has no impact on the wetting properties of the nanocomposite coatings, except at the highest concentration of doped ZnO, i.e., 2.0 wt %, where the contact angle significantly decreases to 99°. Although NiP-C_3_N_4_/2.0 wt % ZnO still exhibits hydrophobic behavior, it is considered more hydrophilic compared to all of the other as-plated coatings, as they reveal contact angles in the range of 100–107°. It is reported in the literature^31,33^ and concluded from BET measurements of the undoped and doped C_3_N_4_, as evaluated in Figure S1, that increasing the concentration of doped ZnO in the C_3_N_4_ nanomaterial significantly increases its porosity, which might eventually influence the compactness of the coating, and hence enhances the surface hydrophilicity. Furthermore, it is noticeable that the coatings become more hydrophilic after heat treatment compared to the corresponding as-plated coatings. As illustrated earlier in XRD and EDX results, new phases form upon heat treatment of the prepared coatings, leading to reduced phosphorous content and increased oxygen. Based on the literature,^38^ the increase in oxygen content is indicative of the formation of an oxide layer on the surface upon heat treatment, which increases the surface wettability. Furthermore, other investigations^39^ reported that the modified structure under heat treatment conditions leads to the increased surface area, which, in turn, increases the wetting area and induces hydrophilic behavior. Regardless of the slight decrease in the surface hydrophobicity after heat treatment, it is important to keep in mind that the differences in the WCA measurements before and after heat treatment are still considered insignificant. Hence, it does not affect the overall performance of the prepared coatings.

### Mechanical Performance Analysis

#### Vicker’s Microhardness Measurements

**Figure 6:**
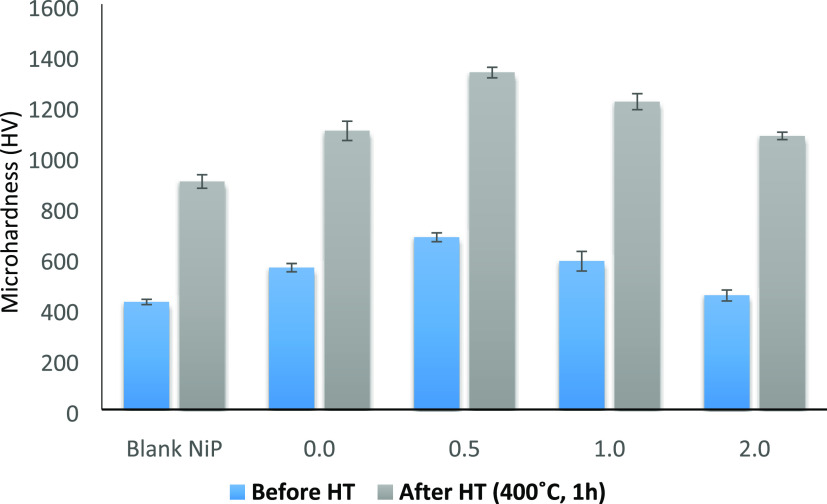
Vicker’s microhardness measurements of the prepared NiP and NiP-C_3_N_4_/ZnO, with different concentrations of ZnO dopant (0.0, 0.5, 1.0, and 2.0 wt %) and nanocomposite coatings before and after HT at 400 °C for 1 h.

The microhardness measurements of the NiP-C_3_N_4_ nanocomposite coatings without and with different concentrations (0.5, 1.0, and 2.0 wt %) of ZnO dopant in comparison with that of the NiP coating, before and after heat treatment, are presented in Figure 6. It can be observed that the incorporation of undoped C_3_N_4_ nanocapsules in the NiP matrix considerably increases its microhardness by about 32%, such that the increase was from 424 to 560 HV_200_, whereas the incorporation of the doped C_3_N_4_ with 0.5 wt % ZnO resulted in a further increase of about 21.4% in the microhardness of the NiP coating, reaching a maximum value of 680 HV_200_. Upon additional increments in the concentration of ZnO dopant in the C_3_N_4_ nanocapsules, namely, 1.0 and 2.0 wt %, the microhardness values of these nanocomposite coatings are decreased to 585 and 450 HV_200_, respectively. However, the microhardness values of these nanocomposite coatings are still higher than those of C_3_N_4_-free coatings. Generally, the increased microhardness values after incorporating undoped and doped C_3_N_4_ nanocapsules could be attributed to the dispersion hardening effect of the nanocapsules that cause stabilizing the dislocations by restricting the grain’s growth and plastic deformation of the coating.^15,40^ Moreover, the highest microhardness obtained for the 0.5 wt % ZnO-doped C_3_N_4_ nanocomposite coating is mainly related to the uniform dispersion of the nanocapsules in the NiP matrix compared to the other concentrations. As reported,^41^ including ZnO nanoparticles in the NiP coating has increased the microhardness value by increasing its concentration to 0.50 g/L. Then, a further increase in the nano ZnO concentration results in decreased NiP composite coating microhardness values.

A significant increase in the microhardness values of the NiP and the undoped, as well as doped C_3_N_4_ nanocomposite coatings, is observed after heat treatment of the specimens at 400 °C for 1 h. The microhardness of the NiP coating increases from 424 to 900 HV_200_, whereas NiP-C_3_N_4_ and NiP-C_3_N_4_/ZnO nanocomposite coatings with different concentrations of ZnO dopant, i.e., 0.5, 1.0, and 2.0 wt %, increase to 1100, 1330, 1215, and 1080 HV_200_, respectively. Based on the obtained results, it can be noticed that the trend of the microhardness values of the nanocomposite coatings before and after heat treatment is generally the same. The microhardness after heat treatment gradually increases until a maximum, which is obtained at the HT NiP-0.5 wt % ZnO-doped C_3_N_4_ nanocomposite coating. Then, it decreases at higher concentrations of ZnO dopant, i.e., 1.0 and 2.0 wt %. The significant increase in the microhardness of HT coatings is mainly related to the formation of a hard Ni__3__P intermetallic phase, which gets more complex and more coherent with Ni at an elevated temperature, i.e., at 400 °C.^42^ Moreover, the presence of undoped and doped C_3_N_4_ nanocapsules in the NiP coating leads to the transition of its phase from amorphous to semicrystalline, as illustrated from XRD results, which becomes crystalline after heat treatment. This provides an extra advantage for increasing the microhardness of HT nanocomposite coatings, especially with the well-dispersed 0.5 wt % ZnO-doped C_3_N_4_ nanocomposite coating. Increased concentrations of ZnO dopant in C_3_N_4_ nanocapsules (2.0 wt %) lead to the aggregation of the nanocapsules even after heat treatment, which, in turn, decreases the microhardness of that nanocomposite coating.

#### Nanoindentation Test

**Figure 7:**
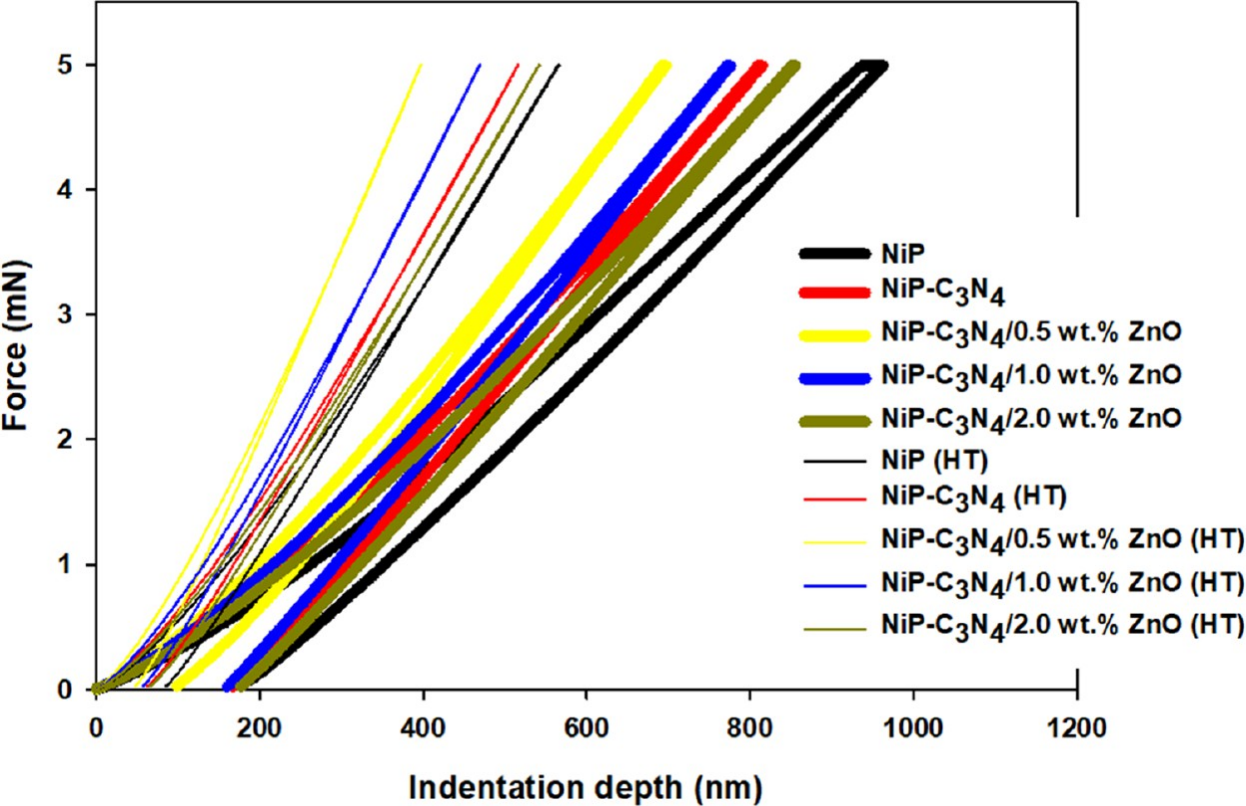
Loading–unloading curves obtained from the nanoindentation test for NiP, NiP/C_3_N_4_, and NiP-C_3_N_4_/ZnO nanocomposite coatings, with different concentrations (0.5, 1.0, and 2.0 wt %) of ZnO dopant, before and after heat treatment at 400 °C for 1 h.

The mechanical hardness of the different coatings was also measured using the nanoindentation technique, which operates with nanometer resolution and a depth in the submicron range. Figure 7 presents the loading–unloading curves obtained from the nanoindentation test applied for the NiP, NiP-C_3_N_4_, and NiP-C_3_N_4_/ZnO composite coatings with different concentrations of ZnO dopant, i.e., 0.5, 1.0, and 2.0 wt %, before and after heat treatment at 400 °C for 1 h. In this technique, as the indentation depth decreases, the hardness of the coating increases. Accordingly, the variations in the coating hardness can be clearly seen through the different indentation depths obtained for both as-plated and heat-treated coatings, as shown in Table 4. Generally, the HT coatings are considered more robust and harder, owing to penetration depths in the range of 48 to 84 nm, whereas the corresponding as-deposited coatings have higher penetration depths in the range of 96 to 190 nm, reflecting their lower hardness values. In addition, it can be noticed that the incorporation of either undoped or doped C_3_N_4_ nanocapsules decreases the indentation depth of the as-plated nanocomposite coatings in the range of 96–183 nm, with hardness ranging from 4.9 to 5.9 GPa, compared to the as-plated NiP coating, which has the highest penetration depth of 190 nm with 4.2 GPa hardness, as shown in Table 4. The smaller displacement in the nanocomposite coatings resulted from the resistance of the NiP matrix to the nanoindenter, showing improved hardness of the coatings upon the addition of C_3_N_4_ nanocapsules. Among the as-plated coatings, the minimum indentation depth is obtained by the 0.5 wt % ZnO-doped C_3_N_4_ nanocomposite coating, indicating the highest hardness (5.9 GPa), which is mainly attributed to the well-dispersed nanoparticles in the NiP matrix compared to the other nanocomposite coatings. In general, the overall improved mechanical performance of the nanocomposite coatings is mainly attributed to the hindered movement of dislocations in the NiP matrix caused by the presence of undoped or doped C_3_N_4_ nanocapsules.^43^ Upon heat treatment, a further increase in the coatings’ hardness is observed, as shown in Table 4, possibly due to the precipitation of the hard Ni_3_P (indicated by the XRD pattern after heat treatment (Figure 1b)). Commonly, various studies^40,44^ have reported the effective increase in the hardness of the NiP and NiP composite coatings after heat treatment upon the formation of the Ni_3_P phase. It is worth mentioning that, as shown in Table 4 and Figure 7, there is an agreement between the results of nanoindentation hardness and Vickers’ microhardness measurements in regard to the pattern and values of microhardness results. For example, the HT 0.5 wt % ZnO-doped C_3_N_4_ nanocomposite coating has the highest microhardness value, which was 10.9 GPa, obtained from the nanoindentation technique, and 1330 HV_200_, obtained from the Vicker’s microhardness, showing that both values are related.

**Table 4:** Penetration Depths (nm) and the Nanoindentation Hardness (GPa) for the Different Coatings

	penetration depth (nm)		nanoindentation hardness (GPa)	
coating name	as-plated	heat-treated	as-plated	heat-treated
NiP	190	84	4.2	8.4
NiP-C_3_N_4_	175	61	5.1	9.8
NiP-C_3_N_4_/0.5 wt % ZnO	96	48	5.9	12.6
NiP-C_3_N_4_/1.0 wt % ZnO	163	58	5.2	10.9
NiP-C_3_N_4_/2.0 wt % ZnO	183	67	4.7	9.5

### Electrochemical Corrosion Analysis

#### Electrochemical Impedance Spectroscopy (EIS)

**Figure 8:**
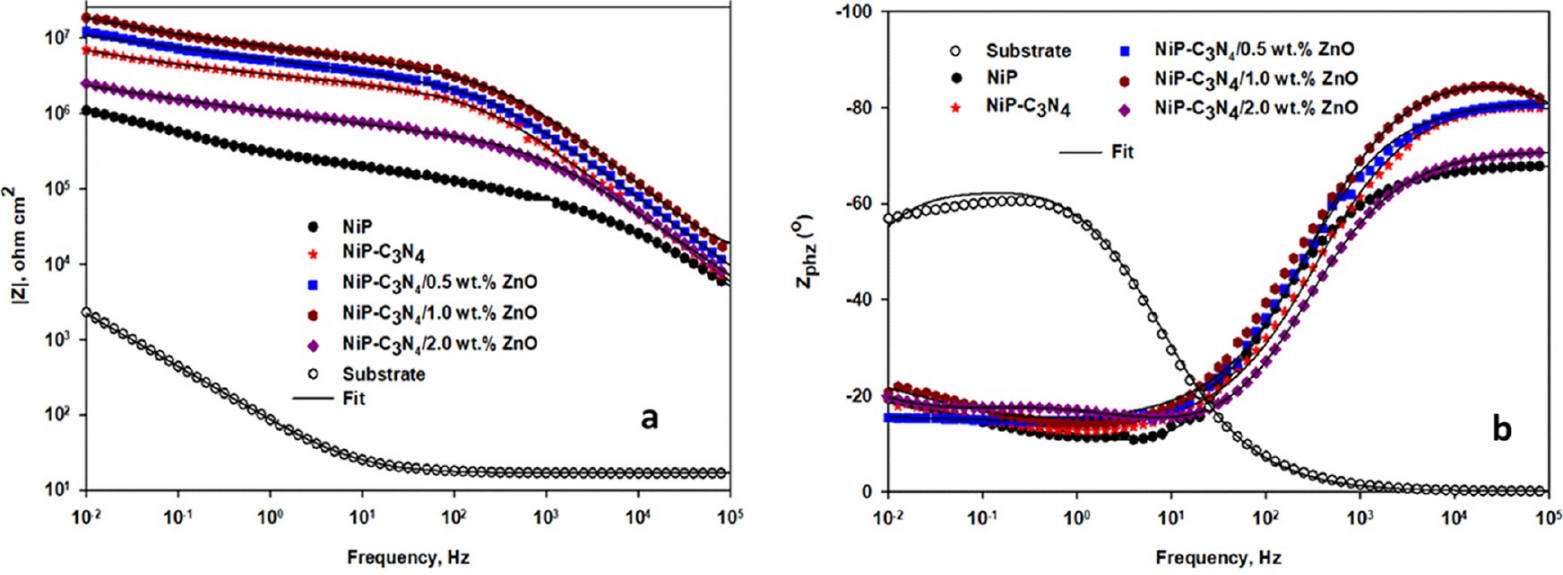
(a) Bode and (b) phase angle plots of the substrate and the as-plated NiP, NiP-C_3_N_4_, and NiP-C_3_N_4_/ZnO nanocomposite coatings, with different concentrations of ZnO dopant in 3.5 wt % NaCl solution at room temperature.

Figure 8a,b, respectively, represents the Bode and phase angle plots obtained from the EIS spectra measured for the substrate C-steel and the as-plated NiP, undoped C_3_N_4_ (NiP-C_3_N_4_), and doped C_3_N_4_ (NiP-C_3_N_4_/ZnO) nanocomposite coatings with different concentrations (0.5, 1.0, and 2.0 wt %) of ZnO. At ambient temperature, the measurements were taken at the open-circuit potential (OCP) in a 3.5% NaCl solution. It is known that in Bode plots, the higher the impedance value at a low frequency, |*Z*_0.01_ Hz|, for the examined sample, the higher its corrosion protection corresponds to lower corrosion rates.^45,46^ As shown in Figure 8a, the |*Z*_0.01_ Hz| value of the as-plated NiP coating is greater than that of the C-steel metal. Upon the incorporation of C_3_N_4_ nanocapsules, either undoped or doped, in the NiP matrix, the |*Z*_0.01_ Hz| values of the as-plated NiP-C_3_N_4_ nanocomposite coatings have generally increased compared to that of the C_3_N_4_-free coating. Furthermore, it can be noticed that increasing the concentration of ZnO in the C_3_N_4_ nanocapsules greatly enhanced the |*Z*_0.01_ Hz| value of the doped C_3_N_4_ nanocomposite coating, where the highest |*Z*_0.01_ Hz| value is obtained for the as-plated 1.0 wt % ZnO-doped C_3_N_4_ nanocomposite coating. However, upon a further increase in the ZnO dopant concentration (2.0 wt %), the |*Z*_0.01_ Hz| value of that coating is significantly decreased, below that of the undoped one. However, it is still higher than that of the C_3_N_4_-free coating. The enhancement in the |*Z*_0.01_ Hz| value of the NiP coating is due to the presence of phosphorous and its reaction with the water forming the hypophosphite layer, which passivates the nickel and protects it from further hydration in the corrosive media.^2^ The further enhancement in the |*Z*_0.01_ Hz| values for the undoped and doped C_3_N_4_ nanocomposite coatings is mainly attributed to the protective strength of the gC_3_N_4_ nanocapsules and the ZnO dopants. Moreover, good distribution of the g-C_3_N_4_ nanocapsules in the NiP matrix had an extra effect in improving coating properties, which became denser with fewer voids and defects, hence reducing the active sites for corrosion attacks. In addition, the increase of ZnO dopant concentration enhanced the compactness of the composite surface, especially at a concentration of 1.0 wt % ZnO as previously demonstrated in the SEM results, which makes it have the highest |*Z*_0.01_ Hz| value. Furthermore, it is worth mentioning that increasing the concentration of ZnO dopant in the C_3_N_4_ nanomaterial significantly increases its surface area and porosity, as evident from the literature^31,33^ and our BET results, which are clarified in Figure S3A and B, as previously mentioned. Consequently, when ZnO was increased to a relatively high amount, the exceedingly increased porosity of the nanocapsules eventually allowed the permeability of the corrosive electrolyte through the coating to the substrate. Additionally, based on TEM measurements for doped C_3_N_4_ nanocapsules shown in Figure S2, the capsule shape of C_3_N_4_ is slightly distorted when it is doped with 2 wt % ZnO becoming oval-shaped, sticky, and highly agglomerated. Hence, it is randomly distributed in the NiP matrix, increasing the agglomeration, as clarified by SEM measurements, leading to reducing the compactness of the composite coating. Therefore, the abovementioned reasons explained the decreased value of |*Z*_0.01_ Hz| for the as-plated 2.0 wt % ZnO-doped C_3_N_4_ nanocomposite coating compared to that of the undoped one. On the other hand, it can be concluded that the protective strength of the 1.0 wt % ZnO-doped C_3_N_4_ nanocomposite coating overpowered its increasing porosity effect in comparison to the 0.5 wt % ZnO-doped C_3_N_4_ one.

Commonly known for assessing a coating’s protective behavior, the high-frequency region is utilized to measure the phase angle, which reflects the capacitive or resistive characteristic.^47^ The literature showed that coatings with high corrosion protection usually have a high phase angle value (θ) at a frequency of 10 kHz.^48^ Obviously, as shown in Figure 8a, the different as-plated coatings have similar shapes and maximal peaks at the high-frequency region, illustrating their protective ability. Nevertheless, there is a considerable variation in their maximal peak values (θ). The 1.0 wt % ZnO-doped C_3_N_4_ nanocomposite coatings have the largest θ value in the high-frequency region compared to the θ values of the other coatings, clarifying its superior protection behavior. The θ values for the different coatings are increased in the order of NiP [65°] > NiP-C_3_N_4_/2.0 wt % ZnO [70°] > NiP-C_3_N_4_ [80°] > NiP-C_3_N_4_/0.5 wt % ZnO [83°] > NiP-C_3_N_4_/1.0 wt % ZnO [89°]. On the contrary, the phase angle plot of the substrate has a different shape compared to the coatings’ samples, and its maximal θ value is at 60°, which appears at the lower frequency side and is lower than the θ values of the different coatings. This indicates a significant high electrical capacitance behavior that leads to a higher corrosion rate^49^ due to the steel surface’s high consumed electrons.

**Figure 9:**
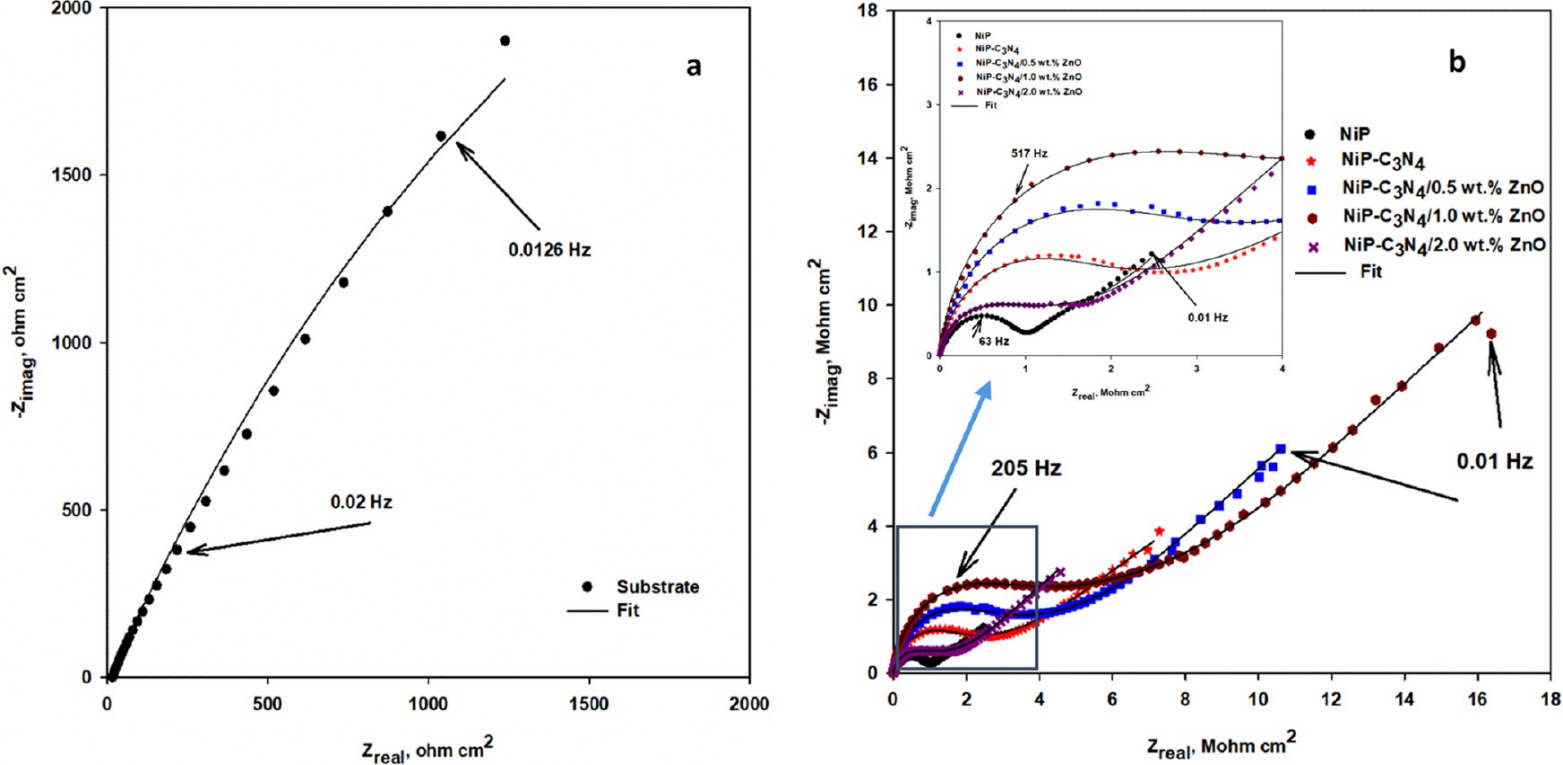
Nyquist plots of (a) the substrate and (b) the as-plated NiP, NiP-C_3_N_4_, and NiP-C_3_N_4_/ZnO nanocomposite coatings, with different concentrations of ZnO dopant, in 3.5 wt % NaCl solution at room temperature. Inset is the enlargement of the low-frequency region.

Figure 9b shows the corresponding Nyquist plots for the as-plated NiP, undoped C_3_N_4_ (NiP-C_3_N_4_), and doped C_3_N_4_ (NiP-C_3_N_4_/ZnO) nanocomposite coatings with different concentrations (0.5, 1.0, and 2.0 wt %) of ZnO in 3.5 wt % NaCl. The magnifications of the different coatings’ low impedance regions of the Nyquist plots can be seen in the inset in Figure 9b. The corresponding Nyquist plot for the substrate can be seen in Figure 9a for a more explicit representation. All Nyquist plots are obtained in the frequency range between 100 kHz–0.01 Hz. The smaller the diameter of the Nyquist semicircle, the smaller the resistive ability of the coating. The Nyquist curves of the different as-plated coatings have the same semicircular shape, which is different from that of the substrate. However, the size and area of the other coatings were considerably different under the Nyquist curves. The similar shape reveals that all of the as-plated coatings undergo the same corrosion mechanism, whereas the different size indicates the different corrosion protection. Therefore, according to the area under the curve for each coating, it is noted that they have the same trend and are consistent with their corresponding Bode plots. For example, the 1.0 wt % ZnO-doped C_3_N_4_ nanocomposite coating has the largest area under its semicircle, clarifying that it has the highest corrosion resistance compared to the other coatings.

**Figure 10:**
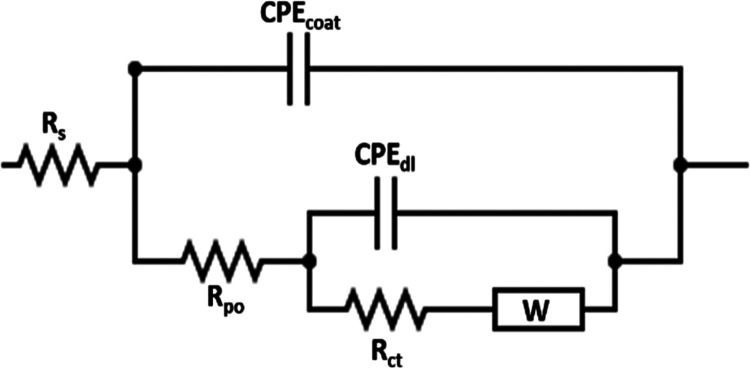
Two-time constant equivalent circuit fits the experimental impedance results of the different as-plated and heat-treated coatings.

The resulting EIS data were fitted using the proper fitting program to analyze the diversity in the impedance spectra of the substrate and the different composite coatings. It is noteworthy that, in Figures 8 and 9, the different colored symbols are the measured EIS data, and the solid black lines represent the fitted data created using the equivalent two-time constant electrical circuit with Warburg diffusion element (*W*), which is shown in Figure 10. The whole fitted parameters are summarized in Table 5. In the equivalent circuit, the *R*_po_, *R*_ct_, and *R*_s_ refer to the pore, charge transfer, and solution resistance, respectively. At a high frequency, i.e., |Z_100_ kHz|, in the Bode plots, the intercept corresponds to the value of *R*_s_, whereas the intercept at a low frequency, i.e., |*Z*_0.01_ Hz|, equals the sum of *R*_po_, *R*_ct_, and *R*_s_. The CPE_dl_ and CPE_coat_ represent the substrate and composite coating constant phase elements, respectively. Finally, the occurrence of electrolyte diffusion is illustrated by the Warburg diffusion element (*W*).^50^ The CPE, a pseudocapacitive element, is utilized to regulate the deviation of the inhomogeneous surfaces, which is obtained due to the roughness or the nonuniform current distribution at the surface^51^ from the ideal capacitive behavior. The impedance value of CPE can be calculated using the following equation^52^
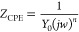
where *Y*_0_ refers to the CPE constant, *j* represents the imaginary number, ω denotes the angular frequency of the AC signal (1/rad), and *n* is the CPE exponent. Its value is fluctuated from 0 to 1 and refers to the state of the working electrode surface. If *n* becomes 1, the CPE displays exemplary capacitor behavior. In addition, the double layer capacitance of the different coatings can be evaluated using the equation below^53^
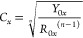
where *Y*_0*x*_ denotes the CPE constant for the coating (*Y*_01_) or the substrate (*Y*_02_) and *R_x_* refers to the pore coating resistance (*R*_coat_) or the charge transfer resistance (*R*_ct_). The substrate/coating interface is demonstrated by the low-frequency time constant, which corresponds to CPE_dl_ and *R*_ct_ as combined. At the same time, the combination of CPE_coat_ and *R*_po_ is related to the high-frequency time constant, which demonstrates the coating/solution interface.

**Table 5:** EIS-Equivalent Circuits Fitting Parameters of the Substrate and the As-Plated NiP, NiP-C_3_N_4_, and NiP-C_3_N_4_/ZnO Nanocomposite Coatings, with Different Concentrations of ZnO Dopant, in 3.5 wt % NaCl Solution at Room Temperature

		CPE_coat_				CPE_dl_				
coating type	*R*_po_ (kΩ·cm^2^)	*Y*_01_ × 10^–9^ (s^n^ ohm^–1^ cm^–2^)	*a*_1_	*C*_coat_ (μF·cm^–2^)	*R*_ct_ (MΩ·cm^2^)	*Y*_02_ × 10^–9^ (s^n^ ohm^–1^ cm^–2^)	*a*_2_	*C*_dl_ (μF·cm^–2^)	*W* × 10^–9^ (S·s^1/2^)	P.E. (%)
CS	3.50	1323	0.89	0.68083	0.005	3094	0.93	2.26071		
NiP	10.2	555	0.76	0.10831	0.90	675	0.76	0.63154	456	99.44
NiP-C_3_N_4_	247	261	0.90	0.19246	10.6	465	0.88	0.57799	365	99.95
NiP-C_3_N_4_/0.5 wt % ZnO	865	157	0.87	0.11650	12.5	339	0.91	0.39104	230	99.96
NiP-C_3_N_4_/1.0 wt % ZnO	960	80.7	0.92	0.06460	22.8	296	0.97	0.31400	189	99.97
NiP-C_3_N_4_/2.0 wt % ZnO	100	277	0.89	0.17781	4.95	843	0.88	1.02431	367	99.87

As noticed in Table 5, the *R*_ct_ value of the NiP coating was about 180 times the *R*_ct_ value of the substrate. This is attributed to the NiP coating surface’s fullness with phosphorus resulting from the dissolution of nickel at the open-circuit potential. Incorporating the undoped or doped C_3_N_4_ nanocapsules, with different ZnO dopant concentrations, in the NiP coating resulted in a significant enhancement in the *R*_ct_ values of their composite coatings compared to that of the C_3_N_4_-free coating. For example, the *R*_ct_ value of the undoped C_3_N_4_ composite coating is increased by about 11.8 times compared to that of the NiP coating. Furthermore, the doping of the C_3_N_4_ nanocapsules with 0.5 wt % ZnO led to an increase in the *R*_ct_ value of the composite coating by 1.2 times. At the same time, doubling the concentration of the ZnO dopant in the C_3_N_4_ nanocapsules, from 0.5 to 1.0 wt %, resulted in almost doubling the *R*_ct_ value of the undoped C_3_N_4_ composite coating. However, a further increase in the ZnO dopant concentration (2.0 wt %) in the C_3_N_4_ nanocapsules minimizes the *R*_ct_ of the composite coating by 47% compared to the undoped C_3_N_4_ composite coating; however, it is still 5.5 times higher than that of the C_3_N_4_-free coating. This is attributed to the good distribution of the undoped or doped C_3_N_4_ nanocapsules in the NiP matrix that leads to an increase in the compactness of the composite coatings, by different degrees, improving their protective performance by diminishing any defects and voids present in the composite coatings. Although the porosity of the doped C_3_N_4_ nanocapsules increases with the increase in the concentration of the ZnO dopant, as proved by the literature and our BET results (Figures S3A and B), the doped C_3_N_4_ composite coatings showed superior barrier performance against the chloride solution, especially with the 1.0 wt % ZnO dopant. This demonstrates its highest protection efficiency reaching up to 99.97%. The higher porosity effect of the doped C_3_N_4_ nanocapsules clearly appears with the highest concentration (2.0 wt %) of the ZnO dopant leading to decreases in the protection efficiency of the composite coating in regard to the undoped C_3_N_4_ composite coating. However, its efficiency is still higher than that of the C_3_N_4_-free coating. Likewise, it is noticed that the pore resistance *R*_po_ values of the different as-plated undoped and doped nanocomposite coatings have the same increasing and decreasing trend as their corresponding *R*_ct_. Furthermore, the embedding of C_3_N_4_/1.0 wt % ZnO nanocapsules in the NiP matrix increases the *R*_po_ value of the resulting NiP-C_3_N_4_/1.0 wt % ZnO nanocomposite coating, as shown in Table 5, where there is up to a 390% increase in comparison with the corresponding *R*_po_ value of the undoped C_3_N_4_ nanocomposite coating (NiP-C_3_N_4_). It is worth mentioning that the different as-plated undoped and doped C_3_N_4_ nanocomposite coatings have lower capacitances (*C*_dl_) and (*C*_coat_) than those of the C_3_N_4_-free coating, and the lowest double layer and coating capacitances values correspond to 1.0 wt % ZnO-doped C_3_N_4_ composite coating. This indicates the efficient impermeability of that coating for the aggressive corrosive ions, hence reflecting its superior protection ability against corrosion.^2,15,25^

**Figure 11:**
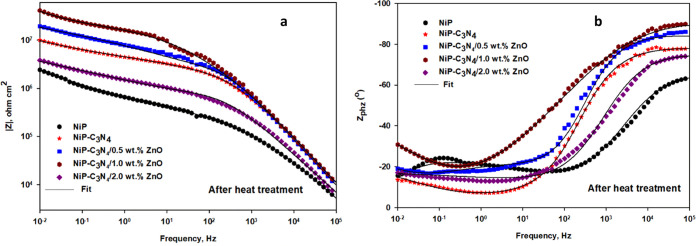
(a) Bode and (b) phase angle plots of the heat-treated NiP, NiP-C_3_N_4_ (undoped), and NiP-C_3_N_4_/ZnO (doped) nanocomposite coatings, with different concentrations of ZnO dopant, in 3.5 wt % NaCl solution at room temperature.

**Figure 12:**
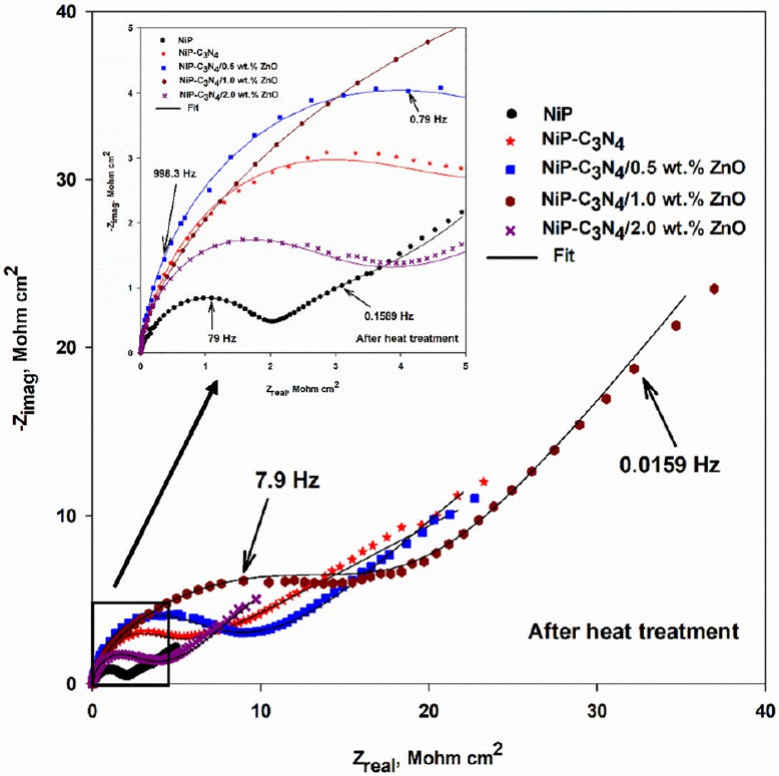
Nyquist plots of the heat-treated NiP, NiP-C_3_N_4_ (undoped), and NiP-C_3_N_4_/ZnO (doped) nanocomposite coatings, with different concentrations of ZnO dopant, in 3.5 wt % NaCl solution at room temperature. The inset is the enlargement of the low-frequency regions.

Figures 11a,b, and 12, respectively, represent the Bode, phase angle, and Nyquist plots, which are measured at the open-circuit potential of the heat-treated NiP, NiP-C_3_N_4_ (undoped), and NiP-C_3_N_4_/ZnO (doped) nanocomposite coatings, with different concentrations of ZnO dopant, in 3.5 wt % NaCl solution at room temperature. The fitted EIS data for the different HT coatings are represented in Table 6. Figures 11a,b, and 12 demonstrate that the Bode, phase angle, and Nyquist plots of the different HT coatings have the same increasing and decreasing trends as their corresponding as-plated ones. For example, in Figure 11a, the |*Z*_0.01_ Hz| of the HT NiP-C_3_N_4_ coating is higher than that of the HT NiP coating. Furthermore, the different heat-treated doped C_3_N_4_ with different concentrations of ZnO dopant has higher |*Z*_0.01_ Hz| than that of the HT-undoped C_3_N_4_ one, and the highest |*Z*_0.01_ Hz| was achieved with the 1.0 wt % ZnO-doped C_3_N_4_ nanocomposite coatings. However, the different HT coatings have higher |*Z*_0.01_ Hz| values than the corresponding as-plated ones. Regarding the phase angle plots of the different heat-treated composite coatings, their maximal peaks (θ) are also in the same order as the corresponding as-plated ones but with a slight increase in their values, as seen in Figure 11b. Thus, the order of increasing θ values is as follows: HT NiP [60°] > NiP-C_3_N_4_/2.0 wt % ZnO [74°] > NiP/C_3_N_4_ [80°] > NiP-C_3_N_4_/0.5 wt % ZnO [86°] > NiP-C_3_N_4_/1.0 wt % ZnO [91°]. Similarly, the Nyquist plots of the different heat-treated coatings have higher semicircle diameters than those of the corresponding as-plated one. On the other hand, all of the corresponding Nyquist curves obtained for the different heat-treated coatings, as presented in Figure 12, revealed the same semicircle shapes but with different sizes and maximal values. Consequently, the same fundamental electrochemical processes have taken place for all HT coatings. Similar to the as-plated coatings, the fitting of the measurements for HT samples followed the two-time constant equivalent circuit with a Warburg diffusion element, as demonstrated in Figure 10. Additionally, the phase angle plots for the different HT coatings at the analyzed frequency range show a two-relaxation process, verifying the two-time constant behavior.

**Table 6:** EIS-Equivalent Circuits Fitting Parameters of the Heat-Treated NiP, NiP-C_3_N_4_, and NiP-C_3_N_4_/ZnO Nanocomposite Coatings, with Different Concentrations of ZnO Dopant, in 3.5 wt % NaCl Solution at Room Temperature

		CPE_coat_				CPE_dl_				
coating type (HT)	*R*_po_ (kΩ·cm^2^)	*Y*_01_ × 10^–9^ (s^n^ ohm^–1^ cm^–2^)	*a*_1_	*C*_coat_ (μF·cm^–2^)	*R*_ct_ (MΩ·cm^2^)	*Y*_02_ × 10^–9^ (s^n^ ohm^–1^ cm^–2^)	*a*_2_	*C*_dl_ (μF·cm^–2^)	*W* × 10^–9^ (S·s^1/2^)	P.E. (%)
NiP	12.5	3.53	0.88	0.00089	5.20	60	0.94	0.05570	360	99.90
NiP-C_3_N_4_	539	2.61	0.80	0.00050	14.3	25	0.93	0.02313	213	99.96
NiP-C_3_N_4_/0.5 wt % ZnO	1147	1.59	0.83	0.00044	18.3	24	0.85	0.02075	153	99.97
NiP-C_3_N_4_/1.0 wt % ZnO	1229	1.51	0.82	0.00037	29.9	9.39	1.00	0.00939	103	99.98
NiP-C_3_N_4_/2.0 wt % ZnO	178	6.03	0.89	0.00259	7.50	47.4	0.88	0.04116	673	99.93

In fact, there was a sharp increase in the corrosion resistance (*R*_po_ and *R*_ct_) of the different coatings after heat treatment, as illustrated in Table 6. This is mainly attributed to the composite coatings’ altered morphology and crystallographic structure upon heat treatment, as previously discussed in the XRD measurements. Furthermore, comparing the fitting parameters for the as-plated and heat-treated coatings (Tables 5 and 6), it can be noticed that the capacitance values, i.e., *C*_coat_ and *C*_dl_, after heat treatment, are decreased compared to those obtained for as-plated coatings. This implies that the HT coatings are denser and less porous than the as-plated ones, preventing the permeability of the corrosive electrolyte ions through the coatings and enhancing their corrosion resistance. It is evident in the literature that the proper heat treatment of the NiP coating can considerably enhance its corrosion resistance as new phases can be formed, inducing a denser and less porous structure.^54^

It is noteworthy that the *R*_po_ and *R*_ct_ resistances, after HT, of the undoped C_3_N_4_ nanocomposite coating are increased by about 118 and 35%, respectively, as compared to their values before HT. In addition, after HT, the *R*_po_ and *R*_ct_ of the coatings improved upon the increase of the ZnO dopant concentration. Therefore, the HT NiP-C_3_N_4_/1.0 wt % ZnO nanocomposite coating offered about 28 and 31% increase in its *R*_po_ and *R*_ct_ values in regard to its corresponding as-plated one, respectively, and about 7 and 63% compared to those of the NiP-C_3_N_4_/0.5 wt % ZnO nanocomposite coating. In addition, as it is previously mentioned, with the as-plated coating, the HT 1.0 wt % ZnO-doped C_3_N_4_ has the highest *R*_po_ and *R*_ct_ values compared to the other HT coatings, followed by a significant decrease in the corrosion resistance *R*_po_ and *R*_ct_ values upon a further increase in the concentration of ZnO dopant. This great enhancement can be related to the excellent compactness and homogeneity of the HT 1.0 wt % ZnO-doped C_3_N_4_ nanocomposite coating, as previously illustrated in the SEM and EDX results.

#### Potentiodynamic Polarization (Tafel Curves)

**Figure 13:**
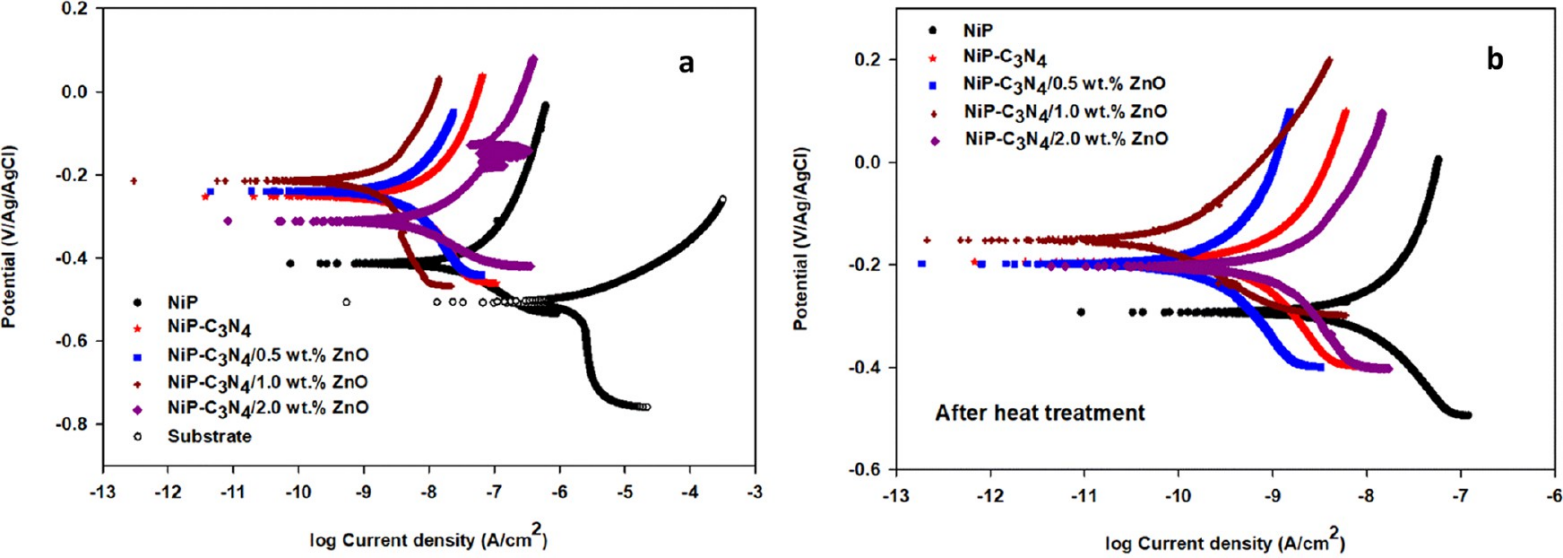
Tafel curves for (a) the as-plated and (b) the heat-treated NiP, NiP-C_3_N_4_ (undoped), and NiP-C_3_N_4_/ZnO (doped) nanocomposite coatings, with different concentrations of ZnO, in 3.5 wt % NaCl solution at room temperature, and the heat treatment at 400 °C for 1 h. The scan rate was 0.167 mV s^–1^.

Figure 13a,b, respectively, shows Tafel plots of the substrate and the as-plated as well as the corresponding heat-treated NiP, NiP-C_3_N_4_ (undoped), and NiP-C_3_N_4_/ZnO (doped) nanocomposite coatings, with different concentrations of ZnO dopant, in 3.5 wt % NaCl solution at room temperature. Table 7 summarizes the various electrochemical parameters: *i*_corr_ (corrosion current density), *E*_corr_ (corrosion potential), *b*_c_, and *b*_a_ (cathodic and anodic Tafel slopes) were acquired using the Tafel extrapolation method and the corrosion inhibition efficiency (I.E. %) for the different as-plated and heat-treated coatings.

**Table 7:** Tafel Fitting Results of the Different Coatings Before and After Heat Treatment

coating	*–E*_corr_ (mV)	*i*_corr_ (nA cm^–2^)	*b*_a_ (V/decade)	*b*_c_ (V/decade)	corro. rate (mpy)	P.E. (%)
CS	500	6890	0.27	0.17	1.5500	
NiP	411	56.0	0.67	0.21	0.0948	99.18
NiP-C_3_N_4_	247	3.40	0.59	0.34	0.0129	99.95
NiP-C_3_N_4_/0.5 wt % ZnO	235	2.30	0.32	0.45	0.0028	99.96
NiP-C_3_N_4_/1.0 wt % ZnO	211	1.20	0.45	0.18	0.0013	99.98
NiP-C_3_N_4_/2.0 wt % ZnO	309	9.20	0.29	0.14	0.0410	99.86
NiP (HT)	289	12.7	0.39	0.49	0.0232	99.81
NiP-C_3_N_4_ (HT)	190	1.02	0.34	0.34	0.0033	99.98
NiP-C_3_N_4_/0.5 wt % ZnO (HT)	192	0.32	0.43	0.11	0.0023	99.99
NiP-C_3_N_4_/1.0 wt % ZnO (HT)	150	0.17	0.19	0.16	0.0001	99.99
NiP-C_3_N_4_/2.0 wt % ZnO (HT)	198	1.30	0.18	0.43	0.0201	99.98

The inhibition efficiency (I.E. %) was calculated using the equation below
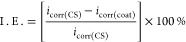
where *i*_corr(CS)_ and *i*_corr(coat)_ correspond to the corrosion current densities for the substrate (API X-120 C-steel) and the coating, respectively.

As demonstrated from the Tafel data, the *E*_corr_ of the substrate is −500 mV, whereas *E*_corr_ for the NiP coating is considerably shifted to the noble direction, reaching −411 mV. In addition, the NiP coating offers a decrease in the *i*_corr_ value about 123 times compared to that of the substrate, indicating good protection ability. Furthermore, all Tafel curves of the as-plated undoped or doped C_3_N_4_ coatings compared to the curve of as-plated C_3_N_4_-free coatings display a successful increase in their *E*_corr_ associated with a decrease in their *i*_corr_, signifying an increase in the corrosion resistance of the NiP coating in the presence of undoped and doped C_3_N_4_ nanocapsules. Furthermore, the *i*_corr_ of the doped C_3_N_4_ nanocomposite coating decreased as the ZnO concentration increased to 1.0 wt %. A further increase in the ZnO concentration (2 wt %) leads to maximizing the *i*_corr_ of the nanocomposite coating by about 7.7 times compared to that doped with 1 wt % ZnO, as shown in Figure 13a and Table 7. Notably, the 1 wt % ZnO-doped C_3_N_4_ nanocomposite coating has the highest corrosion protection efficiency, reaching 99.98%, as shown in Table 7.

The heat-treated NiP, undoped C_3_N_4_, and doped C_3_N_4_ nanocomposite coatings, with different concentrations of the ZnO, showed the same trend as the corresponding as-plated ones, as clarified in Figure 13b. However, their *E*_corr_ is further shifted in the positive direction, and their corrosion current densities were smaller, as shown in Table 7. This indicates that the corrosion resistances of all coatings have significantly enhanced after heat treatment. Furthermore, the decrease in the corrosion rate values of the HT composite coatings confirmed their superior protective ability. This is because of the more compactness of the HT nanocomposite coatings with respect to the corresponding as-plated ones. The *i*_corr_ of the HT NiP is about 77.3% lower than that of the corresponding as-plated one. Noticeably, the doped C_3_N_4_ nanocomposite coating with 1 wt % ZnO has the smallest *i*_corr_ (0.17 nA cm^–2^), displaying a protection efficiency of 99.99%. Finally, it is worth mentioning that the results obtained from potentiodynamic polarization (Tafel analysis) are consistent with EIS outcomes.

### Antibacterial Analysis

Bacterial growth and adhesion on the different surfaces stimulate the biocorrosion of the material, microbiological contamination, economic loss, and healthcare problems. The material surface properties, the type of bacteria, and the surrounding environment govern bacterial adhesion. Therefore, the *staphylococcus* (*S. aureus*) bacterial cell is utilized to evaluate the adhesion and antibacterial activity of the different nanocomposite coatings [NiP, NiP-C_3_N_4_ (undoped), and NiP-C_3_N_4_/ZnO-doped with varying concentrations of ZnO (0.5, 1.0, and 2.0 wt %), before and after heat treatment] using the colony-counting method. For comparison, the substrate (API X-120 carbon steel) is used as a control.

**Figure 14:**
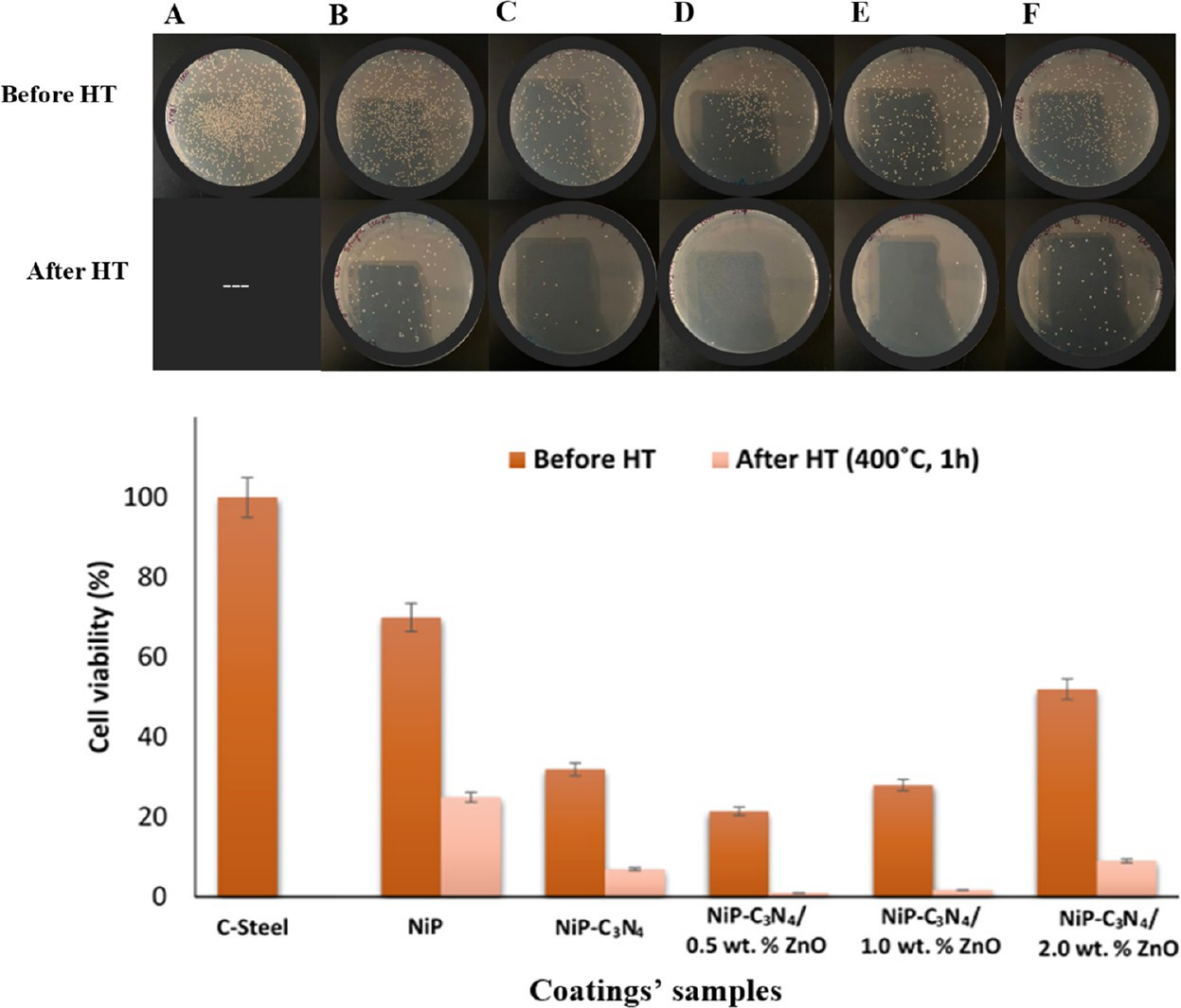
Antibacterial activity of the different coupons. (a) Photographs of *S. aureus* exposed to the substrate (A), NiP (B), and NiP-C_3_N_4_ (C), NiP-C_3_N_4_/0.5 wt % ZnO (D), NiP-C_3_N_4_/1.0 wt % ZnO (E), and NiP-C_3_N_4_/2.0 wt % ZnO (F) nanocomposite coatings. (b) Cell viability (%) of *S. aureus* presents on the different coatings.

Figure 14a depicts the plate photographs of *S. aureus* colony-forming units separated from the different coatings’ surfaces. It is noticed that the as-plated NiP coating has a lower number of *S. aureus* colonies compared to the substrate. Adding undoped (C_3_N_4_) and doped C_3_N_4_ nanocapsules (C_3_N_4_/ZnO) with different concentrations of ZnO into the NiP matrix leads to a further decrease in the number of colonies for the NiP-C_3_N_4_ and NiP-C_3_N_4_/ZnO nanocomposite coatings, respectively, compared to the C_3_N_4_-free coating. Moreover, it is investigated that the increase in the concentration of ZnO maximizes the number of colonies, showing concentration dependence activity. Furthermore, upon heat treatment, the number of *S. aureus* colonies is minimized with all of the different coatings. Figure 14b illustrates the cell viability (%) of *S. aureus* for the different coatings in addition to the substrate. It is observed that the cell viability of the NiP coating is 30% lower than that of the substrate. Additionally, the NiP-C_3_N_4_ and NiP-C_3_N_4_/0.5 wt % ZnO nanocomposite coatings offered an extra 38 and 48.5% decrease, respectively, in cell viability compared to the NiP coating. The rising cell viability of *S. aureus* is noticed with the increasing concentration of ZnO, where the NiP-C_3_N_4_/2.0 wt % ZnO coating shows the highest cell viability compared to the undoped and doped C_3_N_4_ nanocomposite coating with 0.5 and 1.0 wt % ZnO. However, its cell viability is still lower than that of the C_3_N_4_-free NiP coating by 25.7%. The cell viability of *S. aureus* of the different heat-treated coatings has the same increasing and decreasing trends concerning their corresponding as-plated coatings. However, they show a remarkable reduction in cell viability values, reaching 95.3% with the HT NiP-C_3_N_4_/0.5 wt % ZnO coating compared to its corresponding as-plated one. In addition, it is worth mentioning that there is a minuscule change in the cell viability between the HT NiP-C_3_N_4_/0.5 wt % ZnO and NiP-C_3_N_4_/1.0 wt % ZnO coatings, as shown in Figure 14b.

The NiP coating, in particular, plays a significant role in reducing bacterial growth as compared to the substrate. In general, many studies have reported the NiP coating’s bactericidal efficiency.^55−57^ Furthermore, the NiP coating’s antibacterial activity can be due to the Ni^2+^ dissolution from the NiP coating that enters the bacterial cell and inhibits its growth.^58^ A similar mechanism is described for some metals’ antibacterial activity, such as Ag and Zn,^59^ in which metal ion dissolution is essential for antibacterial activity. Dissolved metal ions, physically or chemically bound to the cell wall, can cross the cell membrane and aggregate intracellularly. Metal ions can bind cumulatively to bacterial proteins, making them nonfunctional, leading to the death of the bacterial cell. Furthermore, emerged metal ions could constitute active radicals, resulting in the cell’s death due to its wall breakup.^60^ The substantial reduction in the cell viability of *S. aureus* in the case of as-plated NiP-C_3_N_4_ and NiP-C_3_N_4_/ZnO nanocomposite coatings with different concentrations of ZnO, compared to the NiP coating, confirms the outstanding antibacterial activity of these coatings. This may be attributed to several reasons: (a) when the NiP composite coating is corroded, the C_3_N_4_ nanoparticles become loose. Due to their small size, larger surface area, and active catalytic sites, the loose nanoparticles are more likely to pass through the bacterial cell wall. This penetration leads to changes in the cellular units of the bacteria, leading to its death.^61^ (b) The antibacterial properties of g-C_3_N_4_ and ZnO are assessed and well documented as in refs (62−65). (c) The semicrystalline structure that characterized them, based on XRD results, boosts bacterial death, as in the literature,^66^ and in addition, (d) the hydrophobicity properties of these composite coatings as mentioned in the WCA section. Generally, it is known that hydrophobic surfaces are desired for antibacterial applications.^67^ Therefore, the as-plated NiP-C_3_N_4_/0.5 wt % ZnO and NiP-C_3_N_4_/1.0 wt % ZnO nanocomposite coatings have superior antibacterial properties. On the contrary, the *S. aureus* growth is enhanced on the as-plated NiP-C_3_N_4_/2.0 wt % ZnO coating because of its less hydrophobicity, higher roughness, based on AFM measurements, and less compactness, based on SEM measurements. Furthermore, the less compactness of the NiP-C_3_N_4_/2.0 wt % ZnO coating was also proven by the BET measurements (Figure S1), which show that the increasing concentration of ZnO dopant in the C_3_N_4_ nanocapsules considerably increases its porosity and surface area, as previously mentioned in the WCA section. Moreover, although the different heat-treated coatings show less hydrophobicity and higher roughness, they display outstanding antibacterial behavior because of their smooth and high compactness compared to their corresponding as-plated ones and their crystalline structures, which were obtained upon heat treatment at 400 °C.

Finally, the superior antibacterial properties of the NiP-C_3_N_4_/ZnO nanocomposite coating make it ready to apply in many industries to enhance the anticorrosion performance of several coated materials. In addition, because of the excellent bacterial activity of the NiP coating toward *S. aureus* and other types of bacteria, it is of great importance to conduct more experiments and investigations on this coating. For example, some aspects to be further studied include the relationship between the surface chemistry, roughness, grain size, and microstructure of this antibacterial coating and its composites.

### Deposition Mechanism of Electroless NiP-C_3_N_4_/ZnO Nanocomposite Coating

The extensive work achieved by Gould et al. and other researchers^68^ described that the mechanism of electroless NiP deposition resulted in recent advancements in the electroless NiP coating and its composites in various applications. In general, the reaction kinetics are based on several main steps, starting with the capacities of atomic hydrogen that are formed and adsorbed on the metal’s surface. Then, the nickel ions (Ni^2+^) and hypophosphite ions (H_2_PO_2_^–^) are reduced, resulting in nickel and phosphorus atoms that are codeposited on the surface of the metal. The following equations represent the chemical reactions that occur in the deposition of electroless NiP^69^
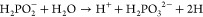

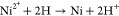

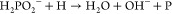
As clarified in the above reactions, the hypophosphite ions (H_2_PO_2_^–^) react with water, producing hydrogen atoms that are desorbed onto the surface of the metal. Next, the ions present in the bath, i.e., nickel (Ni^2^+^^) and hypophosphite (H_2_PO_2_^–^) ions, are reduced by the produced hydrogen atom. Hence, codepositing nickel and phosphorus (Ni–P) are formed. Then, the atomic hydrogen is adsorbed into the formed deposition of Ni–P, and a new Ni and P are codeposited. Finally, the adsorbed atomic hydrogen is consumed, leading to the codeposition of nickel and phosphorus.

**Figure 15:**
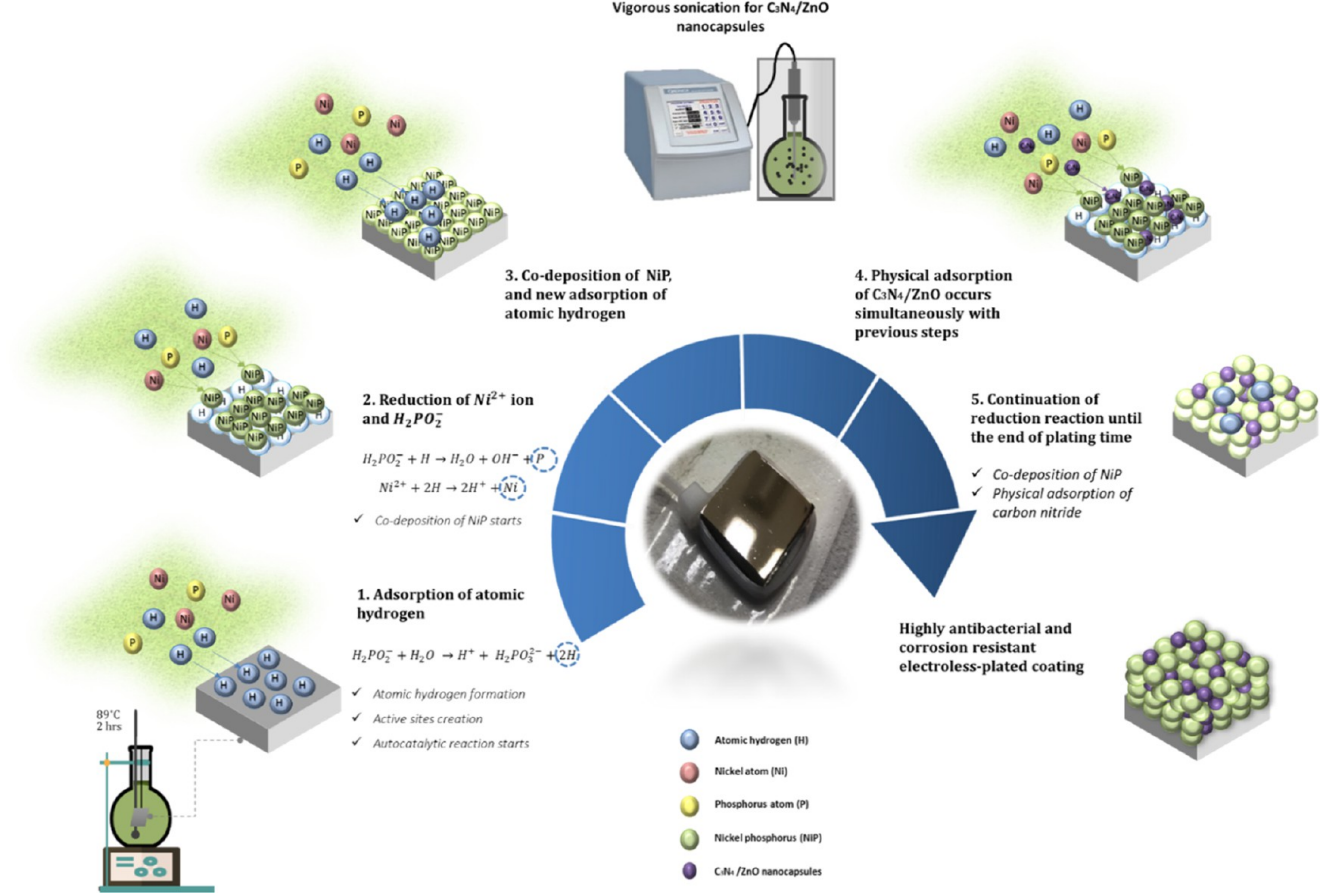
Schematic diagram for the electroless deposition process of the NiP nanocomposites.

The nanocapsules of C_3_N_4_/ZnO undergo a physical adsorption mechanism simultaneously with the deposition mechanism of the electroless NiP coating. The physical adsorption of nanocapsules occurs in two main steps. First, the C_3_N_4_/ZnO nanocapsules, which are well dispersed in the bath, are transported to the electrode surface through mechanical action, and then they are physically adsorbed because of the fluidal attack. Second, due to the chemical adsorption of nanocapsules that occur irreversibly and the electrode’s substantial electric field of Helmholtz layer, the physically adsorbed nanocapsules are dehydrated, followed by covering the adsorbed nanocapsules by the reduced metals or alloys.^70,71^ It is worth mentioning that the agglomeration of C_3_N_4_/ZnO nanocapsules can be reduced through ultra-agitation, which in return improves the overall quality of electroless NiP composite coating. The whole electroless deposition process of the NiP nanocomposites is illustrated in the below schematic diagram (Figure 15).

## Conclusions

Newly capsule-shaped g-C_3_N_4_, either undoped or doped, with different concentrations of ZnO (0.5, 1.0, and 2.0 wt %) as a dopant was successfully incorporated in an orderly distribution inside the NiP coating, which was electroless deposited on API X-120 C-steel. The presence of gC_3_N_4_ nanocapsules does not change the cauliflower-like structure of the NiP coating but leads to the decrease of the size and the increase of the number of its nodules, resulting in a highly compact and crystalline structure. SEM/EDX mapping analyses evidenced the excellent adhesion of the undoped (NiP-C_3_N_4_) and doped (NiP-C_3_N_4_/ZnO) C_3_N_4_ nanocomposite coating on the steel. Moreover, inserting the undoped gC_3_N_4_ nanocapsules in the NiP coating enriched its mechanical, corrosion protection, and antibacterial properties. However, the presence of doped gC_3_N_4_ nanocapsules with a lower concentration of ZnO (0.5 wt %) in the NiP coating significantly enhanced the aforementioned properties of the nanocomposite coatings, offering 99.98% protection efficiency and only 20% *S. aureus* bacterial cell viability with a 21.5% increase in the mechanical properties compared to undoped C_3_N_4_ one. Noteworthily, the properties of all NiP-C_3_N_4_/ZnO nanocomposite coatings have been further enhanced after heat treatment. Therefore, it is highly recommended to utilize these nanocomposites as anticorrosive and antibacterial protective coatings with superior mechanical properties for C-steel, namely, in chloride media.

## Supporting Information Available

BET measurements for undoped and ZnO-doped C_3_N_4_ nanocapsules; TEM analysis for C_3_N_4_/ZnO nanocapsules (PDF)
