# Supplementary material for: ZnO-Doped gC3N4 Nanocapsules for Enhancing the Performance of Electroless NiP Coating—Mechanical, Corrosion Protection, and Antibacterial Properties
Source: ACS Omega. 2023 Jun 13;8(25):22361–81. doi: 10.1021/acsomega.2c07288 (PMC10308405; doi:10.1021/acsomega.2c07288)
Supplement: Supplementary file 1 — ao2c07288_si_001.pdf [file ao2c07288_si_001.pdf]

Supplementary Information

ZnO doped gC_3N_4 nanocapsules for enhancing the performance of electroless NiP coating - mechanical, corrosion protection, and antibacterial properties

Fatma Nabhan[1], Eman M. Fayyad[1,*,a], Mostafa H. Sliem[1], Farah M. Shurrab[2], Kamel Eid[3], Gheyath Nasrallah[2], Aboubakr M. Abdullah[1,*]

[1] Center for Advanced Materials, Qatar University, Qatar, B.OX. 1327

[2] Biomedical Research Center, Qatar University, Qatar, B.OX. 1327

[3] Gas Processing Center, Qatar University, Qatar, B.OX. 1327

[*]Corresponding author: bakr@qu.edu.qa, emfayad@qu.edu.qa

[a] Permanent address: Physical Chemistry Department, National Research Centre, P.O. Box 12622, Dokki, Cairo, Egypt.

Supplementary information 1

BET Measurements for undoped and ZnO-doped C_3N_4 nanocapsules

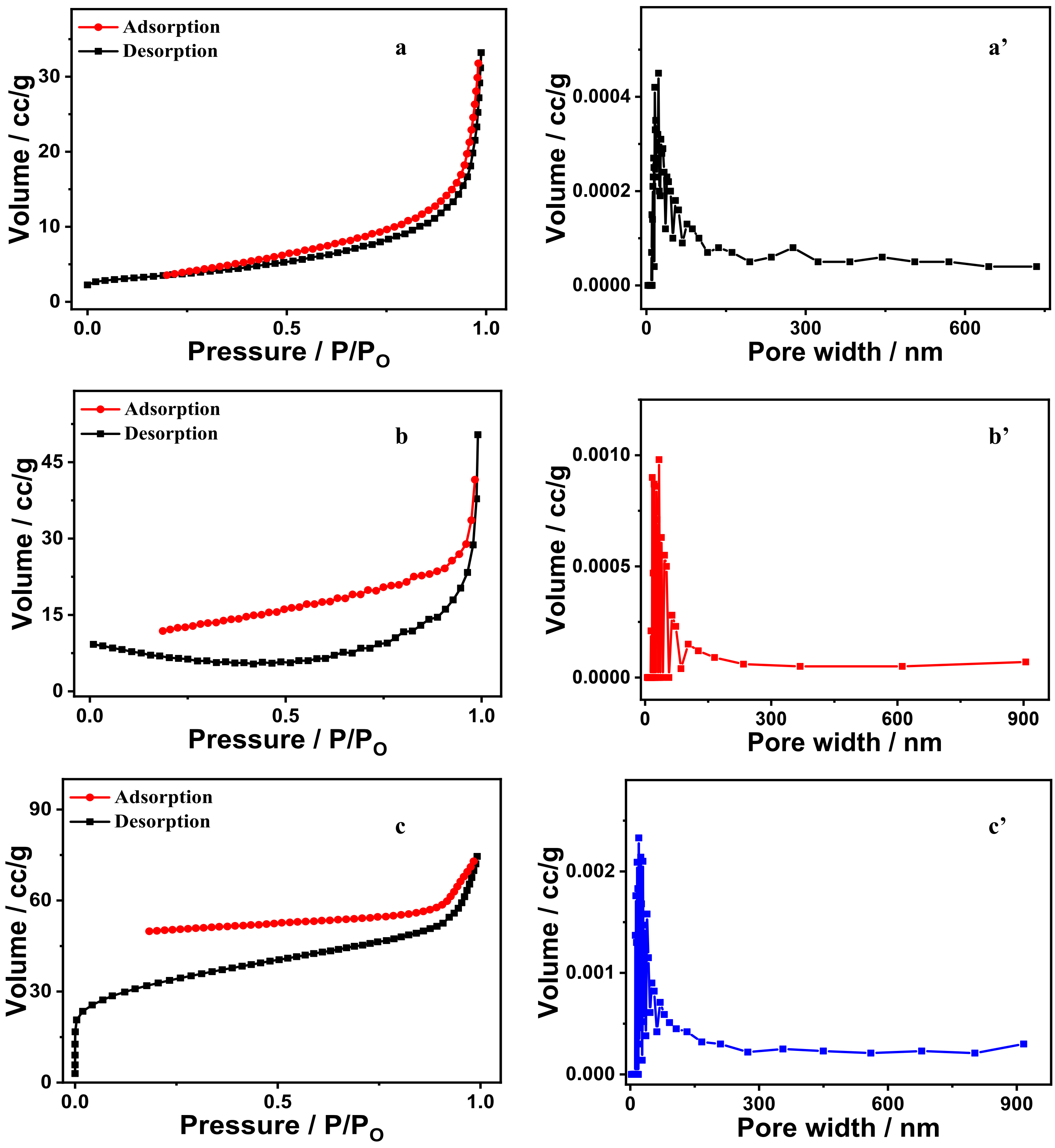

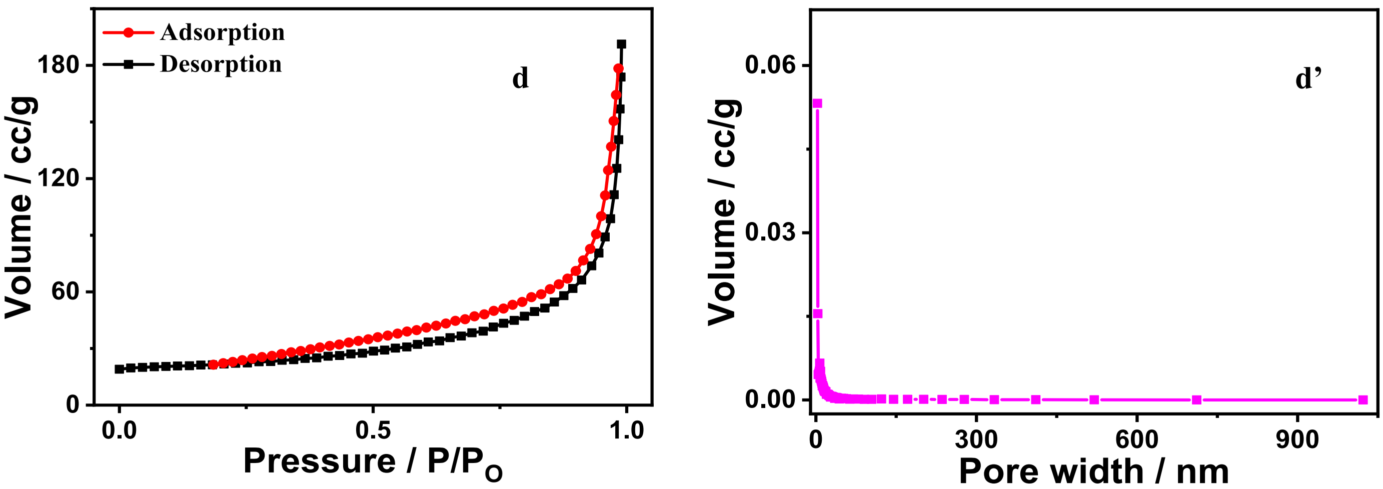

Figure S1. (a, b, c, and d) N2 adsorption-desorption isotherms at 77 K and (a', b', c' and d') Pore size characterization for mesoporous g-C3N4 nanocapsules doped with 0.0, 0.5, 1.0, and 2.0 wt.% ZnO, respectively.

Supplementary information 2

TEM Analysis for C3N4/ZnO Nanocapsules

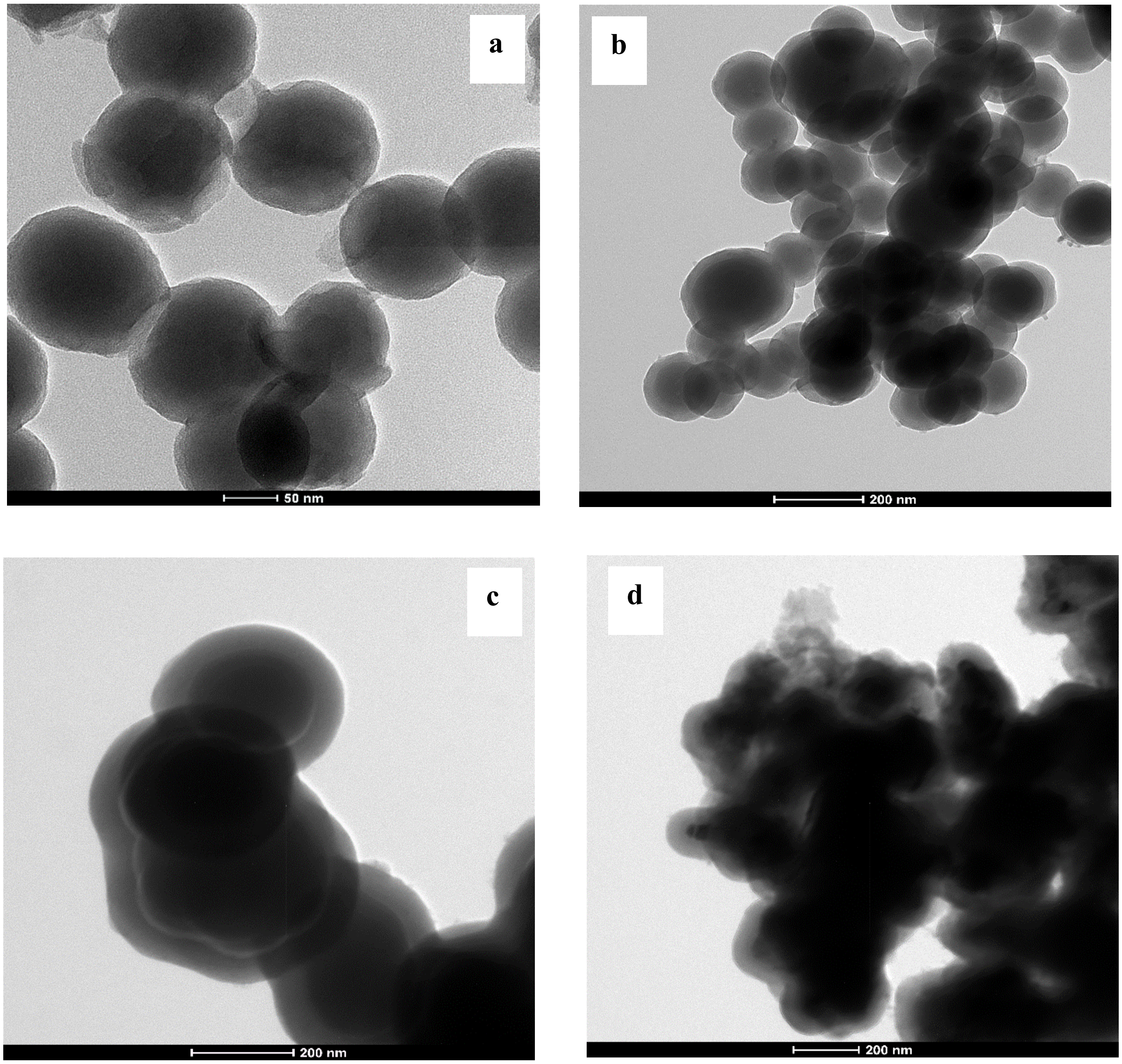

Figure S2. TEM measurements for C3N4 nanocapsules (undoped) (a), and doped C3N4 with (b) 0.5, (c) 1.0, and (d) 2.0 wt.% ZnO.
